# Long non-coding RNAs of switchgrass (*Panicum virgatum* L.) in multiple dehydration stresses

**DOI:** 10.1186/s12870-018-1288-3

**Published:** 2018-05-04

**Authors:** Chao Zhang, Gaijuan Tang, Xi Peng, Fengli Sun, Shudong Liu, Yajun Xi

**Affiliations:** 1 College of Agronomy, Northwest A & F University, Yangling, 712100 Shaanxi China; 2 Key Laboratory of Wheat Biology and Genetic Breeding, Ministry of Agriculture, Yangling, 712100 Shaanxi China; 3 College of Plant Protection, Northwest A & F University, Yangling, 712100 Shaanxi China

**Keywords:** Switchgrass (*Panicum virgatum* L.), Biomass and biofuel, Dehydration stress, Transcriptional profiles, Abscisic acid, Dehydration stress memory

## Abstract

**Background:**

Long non-coding RNAs (lncRNAs) play important roles in plant growth and stress responses. Studies of lncRNAs in non-model plants are quite limited, especially those investigating multiple dehydration stresses. In this study, we identified novel lncRNAs and analyzed their functions in dehydration stress memory in switchgrass, an excellent biofuel feedstock and soil-conserving plant in the Gramineae family.

**Results:**

We analyzed genome-wide transcriptional profiles of leaves of 5-week-old switchgrass plantlets grown via tissue culture after primary and secondary dehydration stresses (D1 and D2) and identified 16,551 novel lncRNAs, including 4554 annotated lncRNAs (targeting 3574 genes), and 11,997 unknown lncRNAs. Gene ontology and pathway enrichment analysis of annotated genes showed that the differentially expressed lncRNAs were related to abscisic acid (ABA) and ethylene (ETH) biosynthesis and signal transduction, and to starch and sucrose metabolism. The upregulated lncRNAs and genes were related to ABA synthesis and its signal transduction, and to trehalose synthesis. Meanwhile, lncRNAs and genes related to ETH biosynthesis and signal transduction were suppressed. LncRNAs and genes involved in ABA metabolism were verified using quantitative real-time PCR, and the endogenous ABA content was determined via high performance liquid chromatography mass spectrometry (HPLC-MS). These results showed that ABA accumulated significantly during dehydration stress, especially in D2. Furthermore, we identified 307 dehydration stress memory lncRNAs, and the ratios of different memory types in switchgrass were similar to those in *Arabidopsis* and maize.

**Conclusions:**

The molecular responses of switchgrass lncRNAs to multiple dehydration stresses were researched systematically, revealing novel information about their transcriptional regulatory behavior. This study provides new insights into the response mechanism to dehydration stress in plants. The lncRNAs and pathways identified in this study provide valuable information for genetic modification of switchgrass and other crops.

**Electronic supplementary material:**

The online version of this article (10.1186/s12870-018-1288-3) contains supplementary material, which is available to authorized users.

## Background

Drought stress is one of the most widespread and harmful abiotic stresses; it includes atmospheric drought and soil drought. Both of these drought types lead to dehydration stress in plants. Under natural conditions, plants suffer thousands of dehydration stresses throughout their lifecycle, and may even experience multiple stresses over a few days. To deal with this situation, plants have evolved dehydration stress memory, which has been identified in *Arabidopsis* [1, 2], maize [3], and other species [4–7]. Using this ability, plants can respond rapidly and strongly to dehydration stress and thus improve their survival rates. The formation of dehydration stress memory involves chromatin methylation (especially histone methylation, such as trimethylation of histone H3 on lysine 4, H3K4me3) [2, 8, 9], plant hormone biosynthesis, and signal transduction [2, 10, 11]. In the *Arabidopsis* genome, distinct regions were found to be susceptible to DNA (de)methylation in response to hyperosmotic stress, and long non-coding RNAs (lncRNAs) regulate the expression of targeted genes in response to stress [12, 13]. Furthermore, animal lncRNAs can interact with trithorax group proteins via H3K4me3 and positively regulate transcription activity [14, 15]. Therefore, we infer that lncRNAs likely have important functions in dehydration stress memory and regulating the responses to multiple dehydration stresses.

LncRNAs are a type of non-coding RNA that are at least 200 nt in length, including natural antisense transcripts, long intronic non-coding RNAs, and long intergenic non-coding RNAs (lincRNAs) [16]. Among plants, a large number of lncRNAs have been found in *Arabidopsis* [17–20], maize [21], wheat [22], rice [23], and other species [24, 25]. LncRNAs play roles in plant growth and development [23, 26–28], biotic stress responses [18, 29, 30], and abiotic stress responses [21, 31, 32]. For example, 2224 lncRNA candidates were discovered in rice, one of which was confirmed to play a role in panicle development and fertility [23]. LncRNA-regulated photoperiod-sensitive male sterility has also been discovered in rice [26]. In *Arabidopsis*, 2708 expressed lincRNAs [20] and 20 *Fusarium oxysporum*-responsive lncTARs (long non-coding transcriptionally active regions) were identified [18], with 245 poly(A) + and 58 poly(A)– lncRNAs differentially expressed under various stress stimuli (drought, salinity, cold, and heat) [17]. Aside from *Arabidopsis* [33] and rice, stress-responsive lncRNAs have also been identified in cotton [25], maize [21], wheat [22], and *Medicago truncatula* [31].

Although numerous lncRNAs have been identified and some of their functions have been described, research in non-model plants has been very limited. The mechanism of lncRNA regulation and its function in dehydration stress are unclear, especially under repetitive dehydration stress conditions. Investigation, functional prediction, and dissection of lncRNAs in response to multiple dehydration stresses will be beneficial for understanding the process of dehydration stress memory, as well as for uncovering the functions and regulatory mechanisms of lncRNAs.

Switchgrass is a perennial, drought-resistant C4 grass in the Gramineae family [34, 35]. It is grown worldwide as an important lignocellulosic biofuel feedstock, soil-conserving plant, and pasture crop [35–37]. Common switchgrass has two ecotypes: the lowland ecotype (tetraploid) and upland ecotype (hexaploid and octoploid) [38]. These ecotypes are allogamous and have strong genetic self-incompatibility, which seriously limits research into biofuel production and stress resistance. In a previous study, we established an in vitro tissue culture system [39]. Using this system, large-scale production of homogenous switchgrass plantlets with a single genotype was carried out from the axillary buds of a single plant. The use of tissue culture avoids the unreliability of cross-pollination and will facilitate future studies of switchgrass.

To date, the majority of studies into drought stress responses of switchgrass are related to its morphological and physiological responses, while the studies on molecular mechanisms of drought resistance have been very limited. Under drought stress, switchgrass improved its drought tolerance by increasing the levels of reactive oxygen species induced by abscisic acid (ABA), causing the water potential, gas exchange rate, and photochemical processes in leaves to decline significantly [40, 41]. Assessment of drought resistance in 49 switchgrass genotypes suggested that drought-tolerant genotypes tend to have higher levels of ABA, spermine, trehalose, and fructose [42]. Furthermore, microRNAs that respond to drought stress were discovered, which were involved in the biosynthesis of carbon compounds, glucose, starch, fatty acids, and lignin [43, 44].

ABA biosynthesis and signal transduction is the key pathway of the drought stress response, involving stomatal closure and osmotic adjustment [45, 46]. In *Arabidopsis*, 9-*cis*-epoxycarotenoid dioxygenase (NCED) catalyzes the rate-limiting step of the ABA biosynthesis pathway [47], and the signal transduction elements include the PYR/PYL receptor, protein phosphatase 2C (PP2C), serine/threonine-protein kinase SRK (SnRK), and ABA-responsive element binding factors (AREBs/ABFs) [48, 49].

LncRNA can play its role through regulating the transcription machinery, as it can directly regulate the Pol II transcription machinery in various ways [15]. LncRNAs located upstream or downstream of genes may interact with promoters, *cis*-acting elements, or other regulatory factors, and thus regulate gene transcription [50–52]. Meanwhile, lncRNA may be involved in gene silencing, transcription, and mRNA stability through complementary base-pairing with the sense strand of mRNA [53]. At present, switchgrass lncRNAs have not been identified, and their functions in drought stress, and especially in repeated drought stress, are unknown.

In this study, switchgrass plantlets of a homogeneous genotype were used for transcriptional and physiological assays. We identified 16,551 novel lncRNAs, and annotated 4554 lncRNAs expressed during multiple dehydration stresses. Functional analysis of the target genes of differentially expressed lncRNAs indicated that the pathways with key roles in repeated dehydration stress response include ABA and ethylene biosynthesis, signal transduction, and starch and sucrose metabolism. These pathways were confirmed using orthologous alignment, quantitative real-time PCR, and chemical assays. Furthermore, we identified dehydration stress memory lncRNAs, and compared dehydration stress memory behaviors among *Arabidopsis*, maize, and switchgrass.

## Methods

### Plant materials and experimental design

**Fig. 1:**
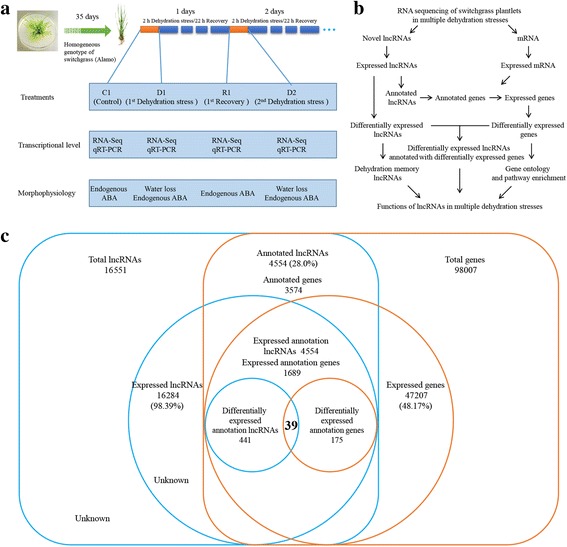
Experimental design and analysis procedures for the systematic identification of lncRNAs in switchgrass. **a** Experimental design for repeated dehydration stresses and sample collection. **b** Functional analysis procedures of lncRNAs in multiple dehydration stresses. **c** Identification and annotation of differentially expressed lncRNAs and annotated genes. ABA, endogenous abscisic acid content; C, control; D1–2, the first and second third dehydration stresses; R1, the first recovery periods; RNA-Seq, RNA-Sequencing; qRT-PCR, quantitative real-time PCR

Switchgrass is allogamous plant with strong genetic self-incompatibility. To avoid differences among seedling genotypes, plantlets developed through in vitro tissue culture were used in this study. They were acquired from a single bud of the Alamo cultivar (introduced from the USA) grown in MS medium supplemented with 13.3 μM·L^− 1^ 6-benzylaminopurine [39]. Multiple dehydration stresses were induced based on designs used previously with *Arabidopsis* [1, 2] and maize [3]. Five-week-old plantlets were removed from the soil, washed of residual substrate, and acclimated overnight in an incubator at constant temperature and humidity with their roots in water. The next morning, the plantlets were removed, patted dry on filter paper, and then air-dried for 2 h (the first dehydration stress, D1). The plantlets under normal conditions were sampled as a control (C1). D1 plants were then placed in a 22-h recovery treatment at 25 °C (first recovery, R1), and R1 plants were subsequently air-dried for 2 h and sampled as the second dehydration stress (D2). These procedures were repeated for R2, D3, etc. (Fig. 1a). To investigate the altered expression of lncRNAs/genes, we sequenced the non-coding RNAs and mRNAs of leaves, and determined the transcriptional expression by qRT-PCR. At morphophysiology level, we determined leaf water loss and endogenous ABA contents in different treatments (Fig. 1a).

### RNA isolation, lncRNA and mRNA library construction

The second fully expanded leaves of plants were collected, frozen immediately in liquid nitrogen and stored at − 80 °C for RNA extraction and physiological and biochemical assays. Total RNA was isolated and purified using Trizol reagent (Invitrogen Life Technologies, USA) and RNase-Free DNase I (Takara, 2270A) following a published protocol [54]. RNA concentration was detected using a Qubit fluorometer and a NanoDrop One spectrophotometer (Thermo Scientific, USA), OD260/280 and OD260/230 (absorbances under 260 nm, 280 nm, and 230 nm ultraviolet light) were detected via a NanoDrop One spectrophotometer, and the RNA integrity (RIN) was measured with an Agilent 2100 bioanalyzer (Agilent Technologies, USA). Two biological replicates of four treatments (C1, D1, R1, and D2) were sequenced, for a total of eight samples. RNA sequencing (RNA-seq) was performed by the BGI-Shenzhen Company (http://www.genomics.cn/en/) using the Illumina GAII platform (HiSeq2500), and lncRNA and mRNA libraries were constructed. To remove adapter sequences and low quality sequences, the raw data was filtered using SOAP software (http://soap.genomics.org.cn/) with the default parameters. Gene and lncRNA expression levels were calculated as FPKM (Fragments Per Kilobase of exon per Million fragments mapped) values using the Cufflinks program [55]. RNA-seq quality and the repeatability of biological replicates are shown in Additional file 1: Figure S1. The raw sequencing files of transcriptomic data were uploaded to the NCBI sequence read archive (SRA) under accession number PRJNA394165.

### Identification of differential expression of genes and lncRNAs

Genes and lncRNAs were predicted based on the switchgrass genomic database (https://phytozome.jgi.doe.gov/pz/portal.html#!info?alias=Org_Pvirgatum) [56]. Differentially expressed genes and lncRNAs were identified using the following threshold values: q value ≤0.05 and | log (base2) fold change | ≥ 1 with the Cuffdiff tool in the Cufflinks package (http://cole-trapnell-lab.github.io/cufflinks/) [55]. Common and differentially expressed genes and lncRNAs in various samples were compared and identified using Venny 2.1 software (http://bioinfogp.cnb.csic.es/tools/venny/index.html).

Dehydration stress memory lncRNAs were identified using methods published for *Arabidopsis* and maize [1, 3]. They were identified by comparison of fold changes in expression between D1/C1 and D2/D1, using threshold values of q value ≤0.05 and | log (base2) fold change | ≥ 1. LncRNAs that were upregulated, downregulated, and those that exhibited no significant change in expression are indicated by the symbols “+”, “–” and “=”, respectively. Eight types of differentially expressed lncRNA were defined: [+/+], [+/−], [−/+], [−/−], [+/=], [−/=], [=/+] and [=/−]. The first four types were considered to be dehydration stress memory lncRNAs, because their responses changed between D1 and D2. The [+/=] and [−/=] lncRNAs were considered non-memory lncRNAs, and [=/+] and [=/−] genes were defined as late-response lncRNAs. A list of all identified dehydration stress memory lncRNAs is provided in Additional file 2: Table S1.

### Annotation and functional analysis of lncRNAs

To date, many lncRNAs located upstream or downstream of genes have been identified, which are involved in transcriptional regulation via interaction with promoters, *cis*-acting elements [50, 51] or other regulatory functions [52]. Meanwhile, lncRNAs may be involved in gene silencing, transcription, and mRNA stability through complementary base-pairing with the sense strand of mRNA [53]. In this study, lncRNAs were annotated by scanning regions within 2000 bp upstream and downstream of genes, and analyzing the complementary base-pairing between antisense lncRNAs and mRNAs using RNAplex software [57].

Genes targeted by lncRNAs were annotated using NCBI Blast tools (version 2.2.8) in conjunction with the NCBI non-redundant protein sequences (nr) database, the *Arabidopsis* Information Resource Proteins database (release TAIR10), the maize B73 RefGen_v3 database, and the Rice Genome Annotation Project (RGAP) release 7. All target genes and differentially expressed target genes were analyzed. Gene ontology (GO) was analyzed using AgBase GORetriever and GOSlimViewer [58], and enriched pathways were identified using NCBI Flink (https://www.ncbi.nlm.nih.gov/Structure/flink/flink.cgi) based on KEGG (Kyoto Encyclopedia of Genes and Genomes) databases. Specific pathways were verified using Blastp with the switchgrass genomic database and the TAIR10 database. Orthologous genes belonging to larger families were analyzed with reference to published works.

### Quantitative real-time PCR, physiological indices assays and statistical analysis

Total RNA was reverse-transcribed to cDNA using the PrimeScript RT reagent Kit (RR047A, Takara) following the standard protocol. Quantitative real-time PCR (qRT-PCR) was performed with the QuantStudio 7 Flex Real-Time PCR System (Thermo Scientific, USA) using the SYBR Premix Ex Taq II Kit (RR820A, Takara). A reported housekeeping gene, *PveEF-1α* (eukaryotic elongation factor 1α) [59], was chosen as a reference gene. Three biological replicates were performed, with each biological replicate having three technical replicates. The ΔΔCT method was used to calculate gene expression levels, and all primers were designed using Primer Premier 6 software (http://www.premierbiosoft.com/primerdesign/index.html; Additional file 2: Table S2).

To verify the ABA biosynthesis pathway indicated by transcriptomic data, the second fully expanded leaves were collected from four treatments. Endogenous ABA contents were determined using high performance liquid chromatography mass spectrometry (HPLC-MS) with an Agilent 1290 Infinity II liquid chromatograph (Agilent Technologies, USA) and a QTRAP 6500 MS/MS System (AB SCIEX, USA). Reference standards (ABA, LC grade) were purchased from Sigma-Aldrich (USA). The water loss was determined by measuring the leaves’ weight at a fixed time interval after their detachment from the plants.

More than three biological replicates were used in all physiological and chemical substance assays. Each biological replicate contained more than 12 plantlets, and only the second fully expanded leaves were utilized for experiments. All data were analyzed with SAS software (http://www.sas.com/en_us/software/sas9.html) with the Duncan test [60].

## Results

### General features of dehydration stress response lncRNAs

Prior to RNA sequencing, we investigated the drought resistance of switchgrass under multiple water-deficit stresses. The results showed that the water loss from leaves during the second and third dehydration stresses was 14.6 and 18.0% slower than during the first dehydration stress after 24 h dehydration treatment, and that water-deficit training significantly improved the survival rates of the plantlets (Additional file 1: Figure S2). In this study, we acquired homogeneous switchgrass plantlets through shoot bud culture, extracted the leaf RNAs under repeated dehydration stresses, and constructed a transcriptional database of lncRNA and mRNA (Fig. 1b). The ratio of clean reads in each sample exceeded 96.5%, and the average replication rate of gene expression numbers in two biological replicates was 90.2% (Additional file 1: Figure S1). For differentially expressed lncRNAs and genes, the average coefficient value between two biological replicates was 99.1% (Additional file 1: Figure S1), indicating excellent repeatability of the results across biological replicates.

**Fig. 2:**
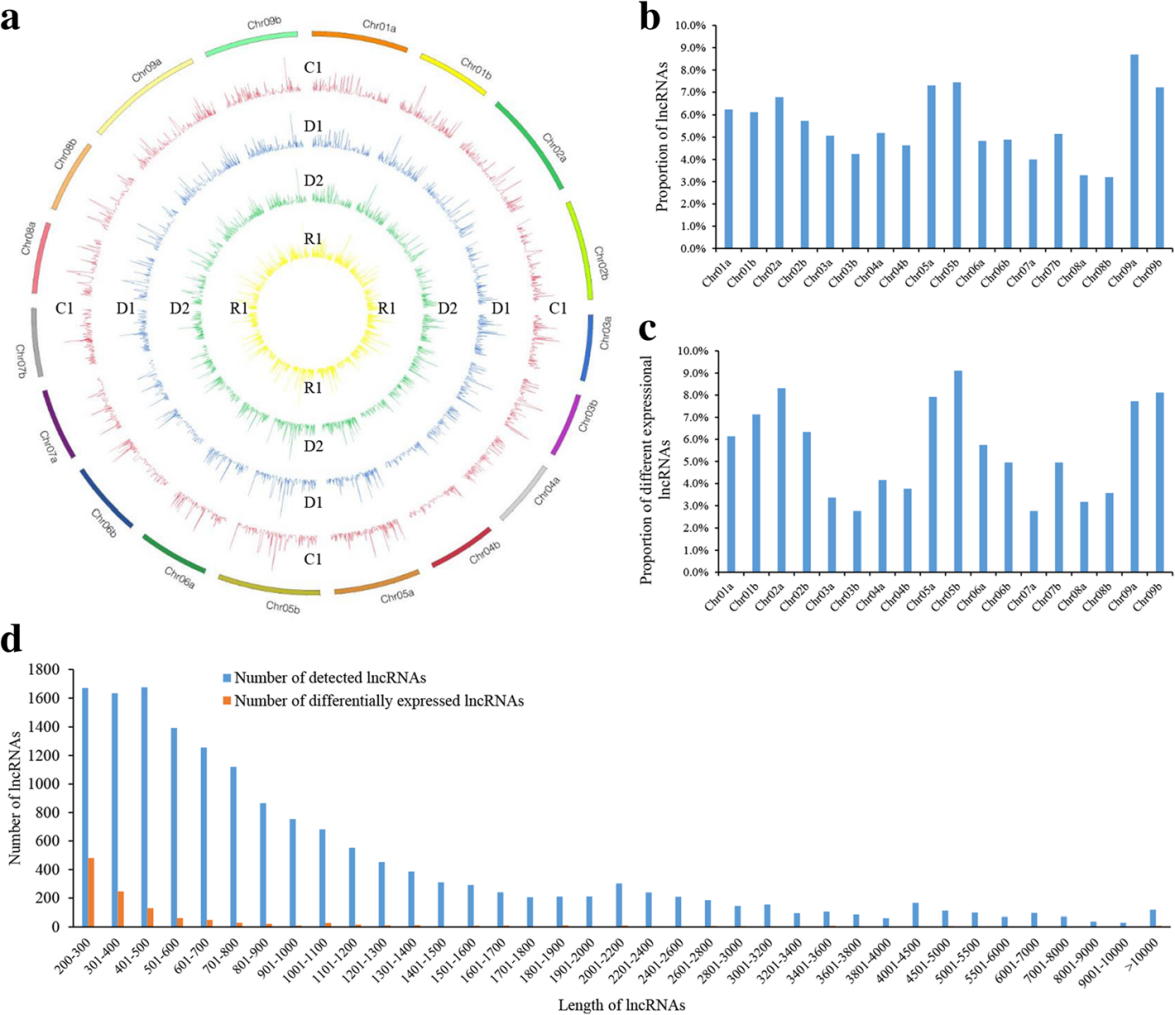
Characteristics of switchgrass lncRNAs. **a** The expression level of lncRNAs (log_10_FPKM) along the switchgrass eighteen chromosomes. It comprises four concentric rings, and they are C1, D1, D2 and R1 from outer to inner, respectively. **b** Distribution of lncRNAs in different chromosomes. **c** Distribution of differentially expressed lncRNAs in different chromosomes. **d** Length distribution of all lncRNAs and differentially expressed lncRNAs. **c**, control; D1–2, first and second dehydration stresses; R1, first recovery period

The expression, location, and length of lncRNAs were analyzed in this study. In total, 16,551 lncRNAs and 98,007 genes were predicted, and 16,284 (98.39%) lncRNAs and 47,207 (48.17%) genes were expressed during dehydration stress (Fig. 1c; Additional file 2: Table S3). In all eight samples, 11,142 (68.4%) lncRNAs and 40,545 (85.9%) genes were expressed. On average, each sample had 19,414 novel transcripts, 38.8% (7549) of which were novel noncoding transcripts (Additional file 2: Table S3). These transcripts were distributed across all chromosomes, and there were some hot-spots in each chromosome with many enriched transcripts (Fig. 2a). We identified differentially expressed lncRNAs using the threshold values: | log (base2) fold change | ≥ 1, q ≤ 0.05. In total, 1597 differentially expressed lncRNAs were identified under repeated dehydration stresses (Additional file 2: Table S4), and they were distributed across all chromosomes, similar to the distribution of total lncRNAs (Fig. 2b, c). Notably, numerous differentially expressed lncRNAs were found on chromosomes 05b, 02a, and 09b (Fig. 2c). In addition, we counted the frequency of different lncRNA lengths. Our results demonstrated that 53.7% of lncRNAs were shorter than 800 bp (base pair), and 59.2% of differentially expressed lncRNAs were shorter than 400 bp (Fig. 2d), suggesting that the lengths of differentially expressed lncRNAs are relatively short.

### Annotation and identification of differentially expressed lncRNAs

LncRNAs located upstream of genes may be involved in transcriptional regulation through interaction with promoters or other *cis*-acting elements [50, 51], and downstream lncRNAs may take part in other regulatory activities [52]. Similar to the action mode of microRNA, lncRNA may be involved in gene silencing, transcription, and mRNA stability through complementary base-pairing with the sense strand of mRNA [53]. In this study, we annotated lncRNAs by scanning up to 2000 bp upstream and downstream of genes, and analyzing the complementary base-pairing between antisense lncRNAs and mRNAs using RNAplex software [57]. In total, 4554 (28.0%) lncRNAs were annotated, including 2129 antisense lncRNAs and 2425 upstream or downstream lncRNAs involved with 2018 and 1556 genes, respectively (Additional file 2: Table S5). During multiple dehydration stresses, 47.3% (1689) of the annotated genes were expressed (Table 1; Fig. 1c). Furthermore, we identified 441 differentially expressed lncRNAs and 175 differentially expressed annotated genes (Additional file 2: Table S6). Among these, 39 lncRNAs were annotated and were differentially expressed genes (Additional file 2: Table S7; Fig. 1c), which probably play important roles in the repeated dehydration stress response. These genes were involved in ABA and ethylene (ETH) biosynthesis and signal transduction, starch and sucrose metabolism, and other functions. (Additional file 2: Table S7).

**Table 1:** Statistic data of annotation lncRNAs and genes

Annotation Type	Number of lncRNAs	Differentially expressed lncRNAs	Annotation genes	Expressed genes	Differentially expressed genes (DEGs)	Differentially expressed lncRNAs annotated with DEGs
antisense-mRNA	2129	142	2018	701	63	13
Up/Down Stream	2425	299	1556	988	112	26
pre-miRNA	Known:9 Novel:47					

### GO and pathway enrichment analysis of lncRNA target genes

**Fig. 3:**
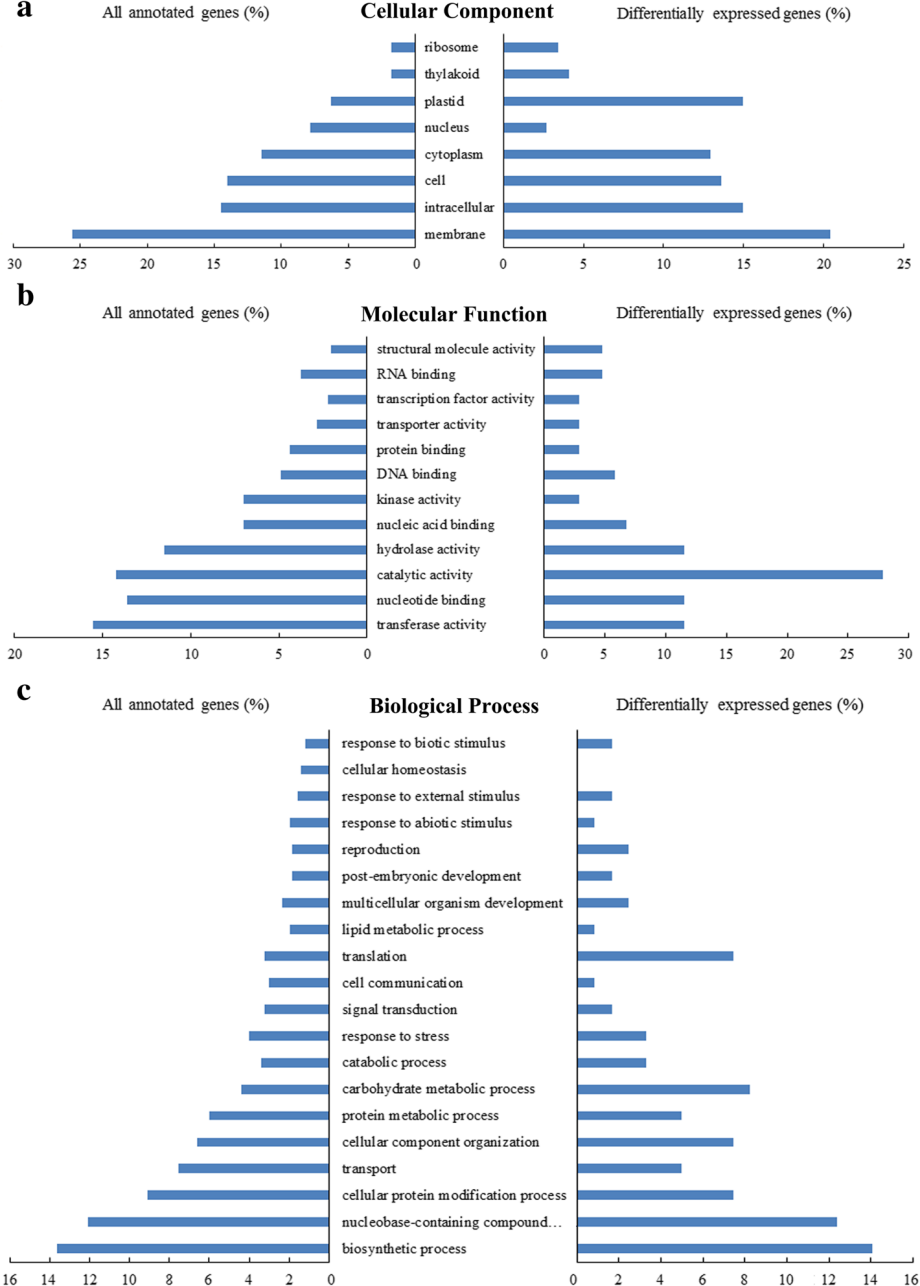
Gene Ontology analysis of lncRNA target genes. **a** Cellular component. **b** Molecular function. **c** Biological process

GO analysis provided information on the functional categorization of lncRNA target genes. We investigated the functions of all annotated genes and differentially expressed annotated genes. The categories of membrane and intracellular (for cellular components) and transferase activity and catalytic activity (for molecular functions) were enriched in all annotated genes, while plastid and catalytic activity were significantly enriched in differentially expressed genes (Fig. 3a, b). For biological process analysis, the terms of biosynthetic processes, nucleobase-containing compound metabolic processes, and cellular protein modification processes were enriched in all annotated genes as well as in differentially expressed genes, while carbohydrate metabolic processes and translation were enriched only in differentially expressed genes (Fig. 3c). The stress response and signal transduction categories were also enriched (Fig. 3c). We analyzed the GO term ‘response to stress’, and identified 15 lncRNAs annotated with 15 genes related to ABA and ETH biosynthesis and signal transduction, starch and sucrose metabolism, osmotic adjustment, and other functions. (Additional file 2: Table S8).

To investigate the roles of lncRNA, we analyzed pathways related to antisense genes, upstream and downstream genes, and differentially expressed annotated genes. The common enriched pathways were biosynthesis of amino acids, plant hormone signal transduction, aminoacyl-tRNA biosynthesis, ribosome, phenylpropanoid biosynthesis, starch and sucrose metabolism, and glycolysis (Additional file 2: Table S9).

Based on these analyses, we find that the ABA, ETH biosynthesis and signal transduction, and starch and sucrose metabolism pathways were significantly enriched in the switchgrass lncRNA annotation, GO analysis, and pathway enrichment during multiple dehydration stresses. These pathways probably play important roles in the dehydration stress response, and we next identified related lncRNAs and genes.

### LncRNAs involved in plant hormone biosynthesis and signal transduction

#### ABA biosynthesis and signal transduction

**Fig. 4:**
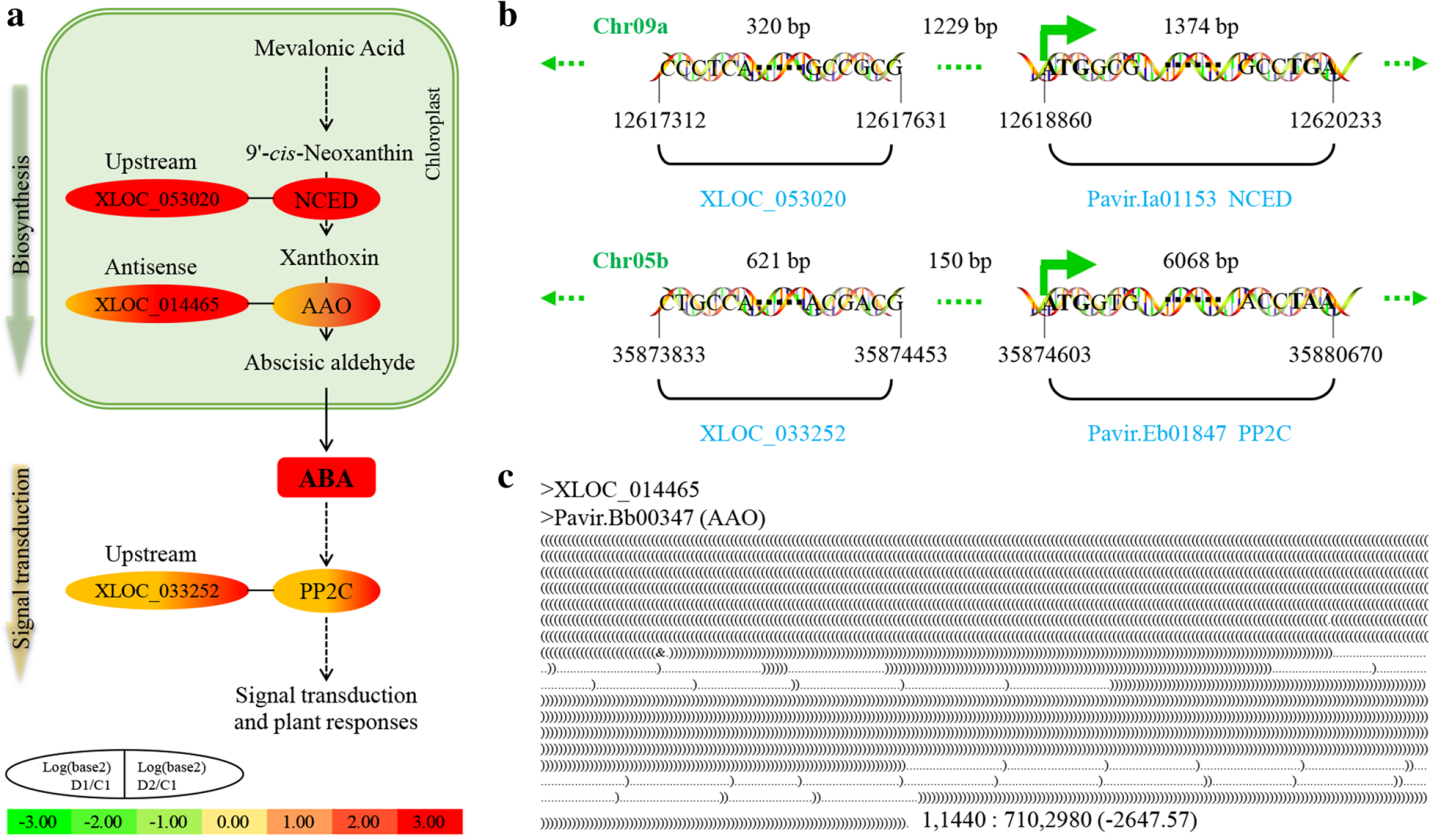
ABA metabolism in multiple dehydration stresses. **a** ABA biosynthesis and signal transduction. **b** Location of differentially expressed lncRNAs. **c** Complementary base-pairing between lncRNA and annotated gene. 1, 1440: 710, 2980 (− 2647.57), complementary start, end for gene: start, end for lncRNA (minimum gibbs energy); ABA, abscisic acid; NCED, 9-cis-epoxycarotenoid dioxygenase [EC 1.13.11.51]; AAO, abscisic-aldehyde oxidase [EC:1.2.3.14]; PP2C, protein phosphatase 2C [EC:3.1.3.16]

ABA biosynthesis and signal transduction were enriched in the differentially expressed lncRNA annotation (Additional file 2: Table S7), GO, and pathway analysis (Additional file 2: Table S7, S8). ABA is the most important plant hormone expressed in response to drought stress, as it is involved in signal perception and transduction, stomatal closure, and osmotic adjustment [11, 46, 61]. In *Arabidopsis*, NCEDs are the key enzymes in ABA biosynthesis, and abscisic-aldehyde oxidases (AAOs) catalyze the last step of this process [47]. During ABA signal transduction, clade A protein phosphatase 2C (PP2CA) enzymes play important roles in ABA signaling and respond positively to ABA level increases or stress-induced ABA biosynthesis [62, 63]. In this study, an lncRNA (XLOC_053020) located 1229 bp upstream of Pavir.Ia01153, an ortholog of *AtNCED3* (AT3G14440, encoding the rate-limiting enzyme of ABA biosynthesis), was upregulated significantly in D1 and D2. Meanwhile, Pavir.Ia01153 was also upregulated (Fig. 4a, b; Additional file 2: Table S10). XLOC_014465 and its antisense gene (Pavir.Bb00347), an ortholog of *AAO1*, were upregulated in D1 and D2. Furthermore, the PP2CA gene Pavir.Eb01847 and its upstream lncRNA XLOC_033252 were both upregulated in D1 and D2 (Fig. 4; Additional file 2: Table S10). These data suggest that ABA biosynthesis and signal transduction were significantly enhanced during multiple dehydration stresses in switchgrass, especially in D2, and the lncRNAs XLOC_053020, XLOC_014465, and XLOC_033252 probably have important functions in stress-induced responses.

#### ETH biosynthesis and signal transduction

**Fig. 5:**
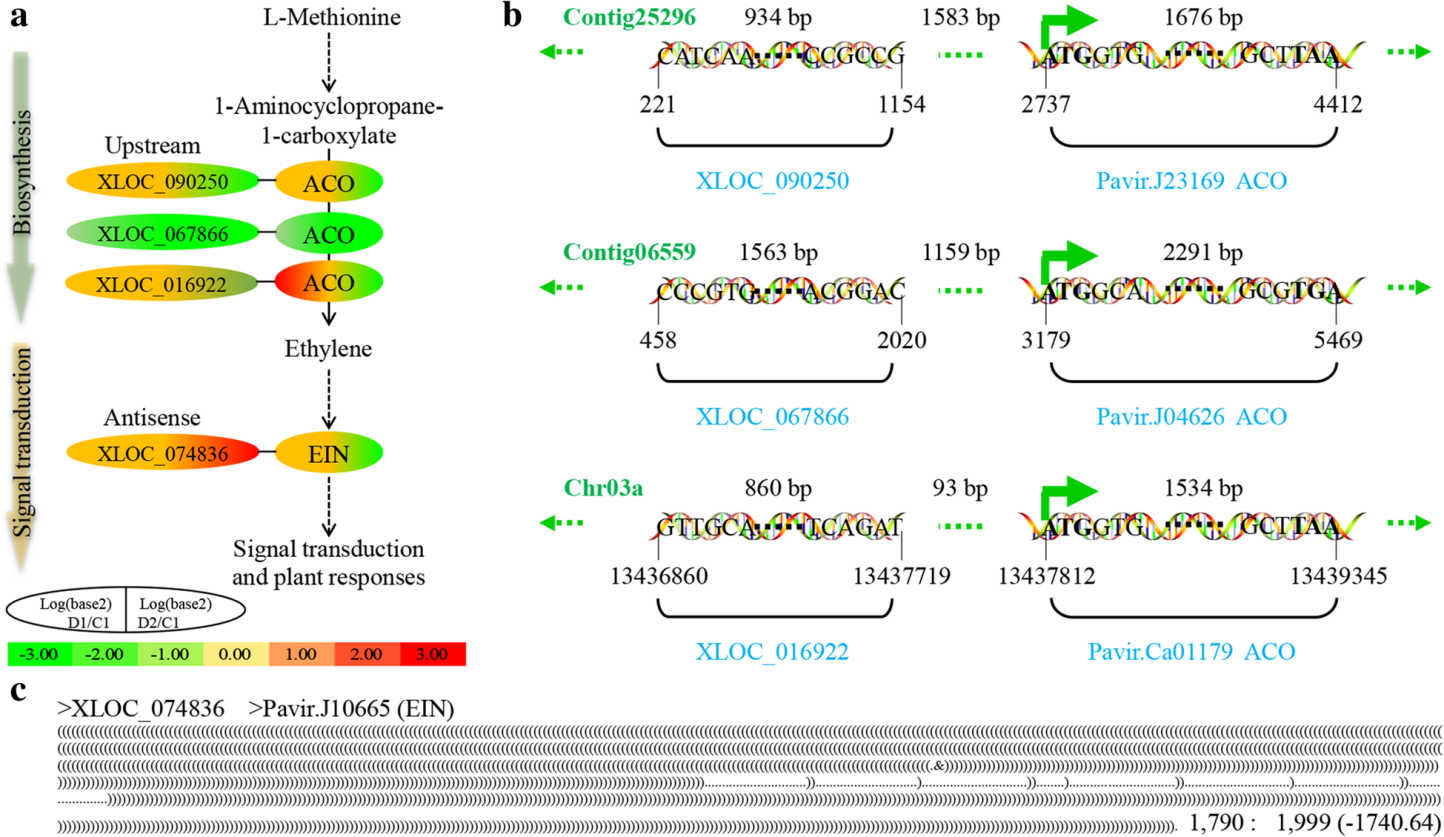
ETH metabolism in multiple dehydration stresses. **a** ETH biosynthesis and signal transduction. **b** Location of differentially expressed lncRNAs. **c** Complementary base-pairing between lncRNA and annotated gene. 1, 790: 1, 999 (− 1740.64), complementary start, end for gene: start, end for lncRNA (minimum gibbs energy); ETH, ethylene; ACO, aminocyclopropanecarboxylate oxidase [EC:1.14.17.4]; EIN, ethylene-insensitive protein

In drought stress, ETH is an important gas hormone, regulating plant growth and senescence [64–66]. In plants, ETH is synthesized from L-methionine, and the rate-limiting enzymes are 1-aminocyclopropane-1-carboxylate synthase (ACS) and aminocyclopropane carboxylate oxidase (ACO) [67, 68]. After synthesis, ETH acts through ETH receptors (ethylene response, ETRs; ethylene response sensors, ERSs; and ethylene insensitive proteins, EINs) [69] and ethylene-responsive transcription factors (ERFs) [70–72]. In this study, two orthologs of *AtACO5* (AT1G77330), Pavir.J23169 and Pavir.Ca01179, were upregulated in D1 and downregulated in D2. Another ortholog of *AtACO4* (AT1G05010), Pavir.J04626, was downregulated in both D1 and D2. Three lncRNAs, XLOC_090250, XLOC_016922, and XLOC_067866, are located upstream of the associated genes. The expression patterns of lncRNAs were similar to those of the related genes (Fig. 5; Additional file 2: Table S11). One gene encoding an EIN-like protein, Pavir.J10665, was upregulated in D1 and downregulated in D2. Its antisense lncRNA, XLOC_074836, was upregulated in D2 (Fig. 5; Additional file 2: Table S11). These data suggest that ETH biosynthesis and signal transduction were slightly upregulated in D1, but significantly downregulated in D2.

### LncRNAs involved in starch and sucrose metabolism

**Fig. 6:**
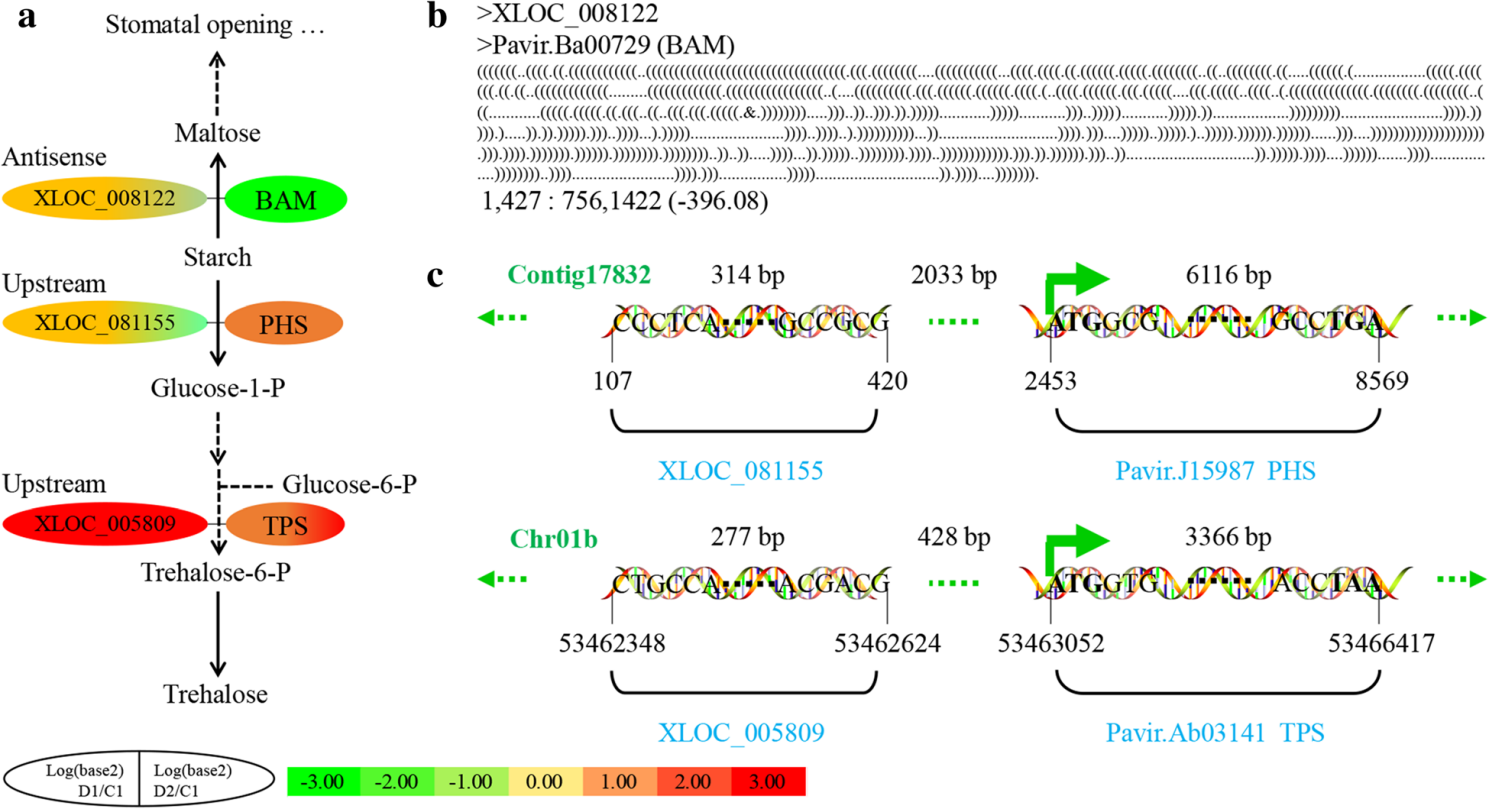
Starch and sucrose metabolism in multiple dehydration stresses. **a** The pathways of starch degradation and trehalose synthesis. **b** Complementary base-pairing between lncRNA and annotated gene. **c** Location of differentially expressed lncRNAs. 1, 427: 756, 1422 (− 396.08), complementary start, end for gene: start, end for lncRNA (minimum gibbs energy); BAM, beta-amylase [EC:3.2.1.2]; PHS, plastidic alpha-glucan phosphorylase [EC:2.4.1.1]; TPS, trehalose 6-phosphate synthase/phosphatase [EC:2.4.1.15 3.1.3.12]

The starch and sucrose metabolism pathway is involved in photosynthesis, energy utilization, and osmotic adjustment. Beta-amylases (BAMs) are important enzymes with roles in starch degradation and sustained stomatal opening [73, 74], which can be transcriptionally induced by heat or cold stress in *Arabidopsis* [75]. In switchgrass under dehydration stress, one ortholog of *AtBAM1/5*, Pavir.Ba00729, was significantly downregulated in D1 and D2, and its antisense lncRNA, XLOC_008122, was also downregulated (Fig. 6; Additional file 2: Table S12). These results indicated that Pavir.Ba00729 was suppressed under water-deficit stress, and therefore suppressed stomatal opening.

Under normal conditions, starch is degraded to glucose-1-phosphate by plastidic alpha-glucan phosphorylase (PHS) [76], and then indirectly converted to trehalose-6-phosphate (T-6-P) and trehalose by trehalose phosphatase (TPP) and trehalose phosphate synthase (TPS), respectively [77]. Trehalose has important roles in osmotic adjustment [46]. T-6-P is implicated in the regulation of sugar metabolism [78], and the genes for its synthesis encode TPSs. In *Arabidopsis*, TPS8–11 belongs to the Class II group, and Class II TPSs are likely regulated by known nutrient signal transduction integrators, such as SnRK1 [77]. In switchgrass exposed to multiple dehydration stresses, an ortholog of PHS was upregulated, and its upstream lncRNA, XLOC_081155, was downregulated in D2. An ortholog of *AtTPS10*, Pavir.Ab03141, and its upstream lncRNA, XLOC_005809, were significantly upregulated, especially in D2 (Fig. 6; Additional file 2: Table S12). These results demonstrate that the starch and sucrose metabolism pathway was altered during dehydration stresses, and that the biosynthesis of T-6-P and trehalose was enhanced, improving sugar metabolism, signal transduction, and osmotic adjustment.

### Verification of lncRNAs and ABA metabolism via quantitative real-time PCR (qRT-PCR) and hormone determination

**Fig. 7:**
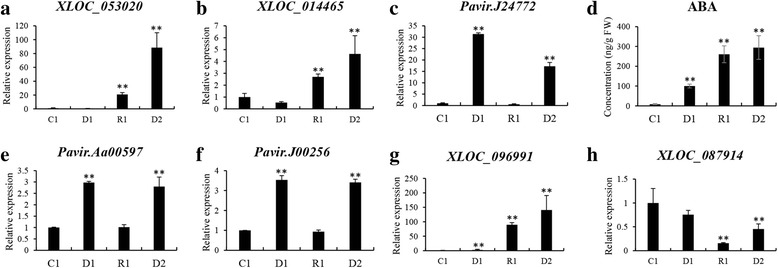
Verification of ABA metabolism and dehydration memory lncRNAs using quantitative real-time PCR and HPLC-MS. **a, b** Relative expression of lncRNAs involved in ABA biosynthesis. **c** Relative expression of ortholog gene of NCED. **d** ABA contents in multiple dehydration stresses determined by HPLC-MS. **e, f** Relative expression of ABA-responsive element binding factors (AREBs/ABFs). **g** Relative expression of upregulated dehydration memory lncRNA. **h** Relative expression of downregulated dehydration memory lncRNA. **c**, control; D1–2, first and second dehydration stresses; R1, first recovery period. Relative gene expressed levels were calculated using the ΔΔC_T_ method with *PveEF-1α* as the internal control, and three biological replicates were performed for each experiment

The RNA-seq results were validated using qRT-PCR in a previous study (Additional file 1: Figure S3). In this study, we quantified the expression of two lncRNAs and three genes involved in ABA biosynthesis and signal transduction, as well as two dehydration stress memory lncRNAs. The expression of all seven transcripts was consistent with transcriptomic data (Fig. 7), indicating that analysis based on transcriptomic data is reliable for determining the response network of switchgrass under multiple dehydration stresses.

We further verified the ABA biosynthesis and signal transduction pathway at the transcriptional and physiological levels. The dynamic change in ABA concentration in leaves was determined using HPLC-MS. The results indicated that ABA accumulated significantly during D1, and increased continuously in R1 and D2 (Fig. 7d). Based on this as well as RNA-seq data and qRT-PCR results, we determined that the upstream lncRNA (XLOC_053020) and orthologs (Pavir.Ia01153 and Pavir.J24772) of the rate-limiting enzyme (NCED), ABA accumulation in leaves, and downstream genes of ABA signal transduction (ABA responsive element binding factor, Pavir.Aa00597 and Pavir.J00256) were upregulated in switchgrass under dehydration stresses, especially in D2 (Fig. 4; Fig. 7; Additional file 2: Table S10). These data indicate that ABA biosynthesis and signal transduction were strongly enhanced during repeated dehydration stresses in switchgrass.

### Dehydration stress memory lncRNAs in switchgrass

**Fig. 8:**
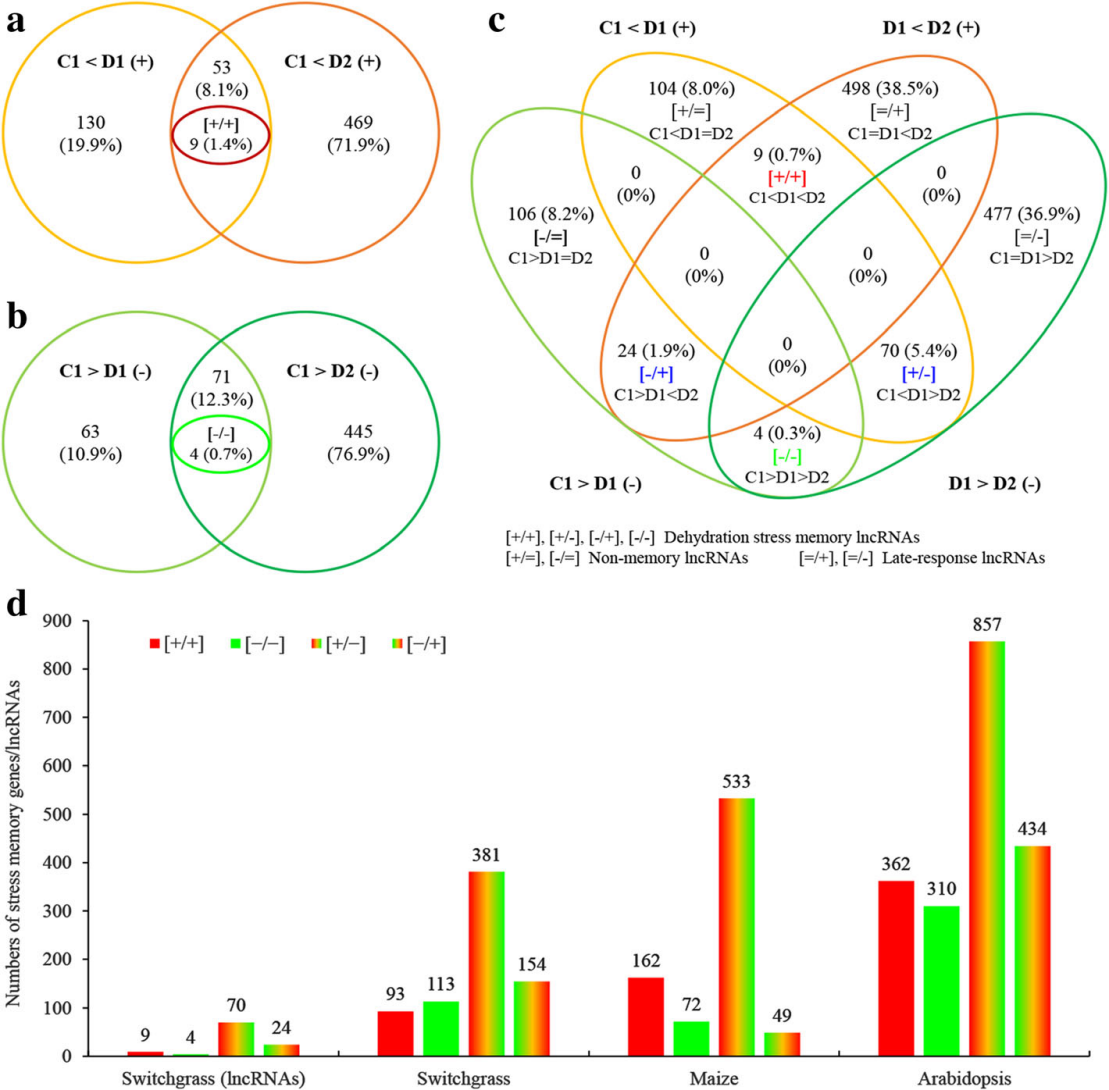
Identification of dehydration memory lncRNAs in switchgrass, and the compare of response numbers to multiple dehydration stress in switchgrass, maize and *Arabidopsis*. **a, b** Venn diagrams of differentially expressed genes in D1 and D2. **c** Venn diagrams of different types of dehydration memory genes. **d** The compare of response numbers to multiple dehydration stress in switchgrass, maize and *Arabidopsis*. **c**, control; D1–2, the first and second dehydration stresses; “+”, “-” and “=” denote up-regulated genes, down-regulated genes, and genes with no significant changes in expression, respectively

During the first round of dehydration stress (D1), 183 and 134 lncRNAs were upregulated and downregulated, respectively, while these numbers increased to 521 and 516 in the second dehydration stress (D2) (Fig. 8a, b). This result indicated that the dehydration stress responses in D1 and D2 differed greatly. In this study, we identified dehydration stress memory lncRNAs in switchgrass using screening methods for dehydration stress memory genes reported in *Arabidopsis* and maize [1, 3]. These genes were identified through comparison of fold changes in expression between D1/C1 and D2/D1. LncRNAs that were upregulated, downregulated, and exhibited no significant change in expression are indicated by the symbols “+”, “–”, and “=”, respectively. Nine lncRNAs were identified as [+/+] memory type lncRNAs, meaning that these lncRNAs were upregulated between D1 and C1 and also between D2 and D1 (Fig. 8c). Similarly, the numbers of [+/−], [−/+], and [−/−] lncRNAs were 70, 24, and 4, respectively (Fig. 8c, d). Most dehydration stress memory lncRNAs (76.6%) were not annotated, and thus their functions remain unknown (Additional files 2: Table S1).

## Discussion

Under natural conditions, plants are frequently subjected to dehydration stress due to vapor pressure deficit or soil drought. They are exposed to repeated dehydration stresses throughout their lifecycle, often in quick succession. When the quantity of water lost from leaves through transpiration is greater than the water taken up, plants will suffer from dehydration stress during the daytime. At night, they will recover, but may suffer from dehydration stress again the next day. In the process of evolution, plants have developed dehydration stress memory to adapt to repetitive dehydration stresses. This phenomenon was discovered in *Arabidopsis* [1, 2], then identified in maize [3]. In stress memory, plants improve their survival rates by rapidly and strongly responding to dehydration stress. Previous studies showed that dehydration stress memory is related to chromatin methylation (especially histone methylation) [2, 8, 9], and lncRNA plays important roles in chromatin methylation and dehydration stress responses [12, 13, 21, 31]. Therefore, we imitated the diurnal cycle of dehydration stress in this study, then constructed lncRNA and mRNA libraries, and annotated, identified, and verified lncRNAs involved in multiple dehydration stresses.

LncRNA was annotated by scanning 2000 bp upstream or downstream of genes based on the switchgrass genomic database [50–52], and then analyzing complementary base-pairing between antisense lncRNAs and mRNAs using RNAplex software [57]. In this study, 4554 lncRNAs were annotated, and 9.7% (441) of these were differentially expressed (Table 1; Fig. 1c). We analyzed the functions of the predicted target genes of all lncRNAs as well as differentially expressed lncRNAs. GO and pathway enrichment analysis showed that ABA and ethylene biosynthesis, signal transduction, and starch and sucrose metabolism were significantly enriched under repetitive dehydration stresses (Fig. 3; Additional file 2: Table S9). In addition, when the above pathways were analyzed, we found that most functional lncRNAs were located upstream of genes, and 85.7% had similar expression patterns with their downstream genes (Figs. 4–6; Additional file 2: Tables S10–12). The sequence 2000 bp upstream of a target gene is the promoter region, and therefore we inferred that these lncRNAs functioned by interacting with the promoter, *cis*-acting elements, or other regulatory activities, and thus influenced the transcription of downstream genes [15, 50–52].

To date, ABA is considered the most important hormone involved in plant drought stress responses, and plant drought resistance can be improved significantly by increasing ABA biosynthesis or signal transduction, such as enhancing ABA receptors [79, 80]. In our study, the orthologs of the rate-limiting enzyme of ABA biosynthesis, Pavir.Ia01153 and Pavir.J24772, were significantly upregulated in D1 and D2 (Figs. 4, 7). The lncRNA XLOC_053020, located 1229 bp upstream of Pavir.Ia01153, was upregulated in D1 and D2 (Figs. 4, 7; Additional file 2: Table S10). The signal transduction factors ABF (Pavir.Aa00597, Pavir.J00256) and PP2C (Pavir.Eb01847) were also upregulated during repeated dehydration stresses. The lncRNA XLOC_033252, located 150 bp upstream of Pavir.Eb01847, has the same expression pattern as Pavir.Eb01847 (Fig. 4). The expression levels of these key genes and lncRNAs were verified using qRT-PCR, and the dynamic change in ABA levels was also determined with HPLC-MS (Figs. 4, 7). These data demonstrated that ABA biosynthesis and signal transduction were significantly enhanced during multiple dehydration stresses, and thus the related lncRNAs likely played important roles in these processes. Enhanced ABA metabolism will result in changes in stomatal closure, osmotic adjustment, or metabolic pathways [46, 61].

In contrast to ABA metabolism, ETH biosynthesis and signal transduction were weakened, especially in D2. In *Arabidopsis*, many key enzymes and factors of this pathway respond to hyperosmotic stress, such as ACOs, ACSs, ERFs, RTRs, ERSs, and EINs. For example, *TaACO1* (wheat) negatively regulates salinity stress in *Arabidopsis* [81], and three ACO genes, two ETR genes, and four EIN genes were significantly upregulated in physic nut leaves under severe drought stress [82]. In this study, three ACO ortholog genes and one EIN ortholog were downregulated in D2, three lncRNAs located upstream of ACO genes were also downregulated in D2, and one antisense lncRNA of an EIN gene was upregulated in D2 (Fig. 5; Additional file 2: Table S11). These results indicated that ETH biosynthesis and signal transduction were downregulated in D2, demonstrating that switchgrass plants adapted rapidly to repeated dehydration stress in D2. The decrease in ETH is beneficial to plant growth and suppresses leaf senescence [66, 83]. In addition, the differential expression patterns of upstream and antisense lncRNAs with target genes indicated differing action modes of upstream lncRNAs and antisense lncRNAs.

Aside from the plant hormone biosynthesis and signal transduction pathways, starch and sucrose metabolism were also enriched in the functional analysis of lncRNA target genes (Fig. 3; Additional file 2: Table S9). Starch can be degraded to maltose by beta-amylases, and sustains stomatal opening [73, 74]. In this study, an ortholog of beta-amylase was significantly downregulated in D1 and D2 (Fig. 6; Additional file 2: Table S12), which would promote stomatal closure. On the other hand, starch can be degraded to glucose-1-phosphate by PHS [76], and then indirectly converted into T-6-P and trehalose by TPP and TPS, respectively [77]. In switchgrass under dehydration stress, orthologs of PHS and TPS were significantly upregulated in D1 and D2, and one lncRNA located 428 bp upstream of the TPS gene was also significantly upregulated. These results suggest that starch metabolism was affected and trehalose biosynthesis was enhanced under dehydration stress, which greatly improved carbohydrate metabolism and osmotic adjustment [46].

Following identification and functional analysis of lncRNAs, we sought transcriptional memory lncRNAs expressed under repetitive dehydration stresses. Using a method and threshold values reported for *Arabidopsis* and maize [1, 3], we identified 107 dehydration memory lncRNAs, including 9 upregulated memory ([+/+]) lncRNAs, 4 downregulated memory ([−/−]) lncRNAs, 70 [+/−] memory lncRNAs, and 24 [−/+] memory lncRNAs (Fig. 8c, d; Additional file 2: Table S1). Remarkably, the number of [+/−] lncRNAs (65.4%) was significantly greater than the other three types, and this phenomenon is apparent in the ratios of dehydration stress memory genes in switchgrass (51%), *Arabidopsis* (44%), and maize (65%) (Fig. 8c, d; Additional file 2: Table S13). The mechanism of this phenomenon is unknown, and 76.6% of the dehydration stress memory lncRNAs were not annotated, and thus their functions remain unknown (Additional file 2: Table S1). High-throughput RNA sequencing may allow elucidation of dehydration memory genes and lncRNAs, and the information acquired in this study provides a strong foundation for analysis of the sophisticated network.

## Conclusions

In this study, switchgrass plantlets of a homogenous genotype were exposed to multiple dehydration stresses. We identified 16,551 lncRNAs, of which 441 were differentially expressed. We annotated 4554 lncRNAs in up/downstream or complementary base-pairing analysis, while GO and pathway enrichment analysis demonstrated that these lncRNAs were involved in ABA and ETH biosynthesis and signal transduction, as well as starch and sucrose metabolism. Subsequent analysis indicated that ABA and trehalose were synthesized significantly in D1 and D2, and ABA signal transduction was also enhanced. Meanwhile, ETH biosynthesis and signal transduction was suppressed. Transcriptomic data, including genes involved in ABA metabolism and the associated lncRNAs were verified using qRT-PCR. HPLC-MS showed that ABA accumulated significantly during dehydration stress, especially in D2. Furthermore, 307 dehydration stress memory lncRNAs were identified and we found that the ratios of different memory types are similar in switchgrass, *Arabidopsis*, and maize. In this study, we identified and characterized lncRNAs in switchgrass repetitive dehydration stress, and the lncRNAs and genes identified in this paper provide novel resources for genetic modification of switchgrass and other crops in the Gramineae family, in particular wheat, rice, and maize.

## Additional files

Additional file 1: **Figure S1.** Overview of the RNA-Seq results and repeatability of different biological replicates. The first eight figures show the proportion of clean reads in the sequenced samples; the next three figures show the repeated expression of genes in two biological replicates; the last eight figures showed the correlations between differentially expressed genes in two biological replicates, and the distributions of upregulated and downregulated genes/lncRNAs in multiple dehydration stresses. C, control; D1–2, the first and second dehydration stresses; R1, the first recovery period. **Figure S2.** Water loss and survive rates in multiple dehydration stresses. A. Water loss from leaves during the first, second and third dehydration stresses. B. Survival rates of trained and non-trained switchgrass. **Figure S3.** Verification of four types of dehydration memory genes by quantitative real-time PCR. C, control; D1–2, first and second dehydration stresses; R1, first recovery period. Relative gene expressed levels were calculated using the ΔΔCT method with *PveEF-1α* as the internal control, and three biological replicates were performed for each experiment. (ZIP 1905 kb)

Additional file 2: **Table S1.** List of memory lncRNAs. **Table S2.** Primers used in qRT-PCR. **Table S3.** Expression summary of different samples and treatments. **Table S4.** List of differentially expressed lncRNAs. **Table S5.** LncRNAs annotated by antisense and upstream or downstream. **Table S6.** Differentially expressed lncRNAs and the annotated genes. **Table S7.** Differentially expressed lncRNAs annotated with differentially expressed genes. **Table S8.** GO term _ response to stress. **Table S9.** Top 20 pathway enriched in annotation genes. **Table S10.** LncRNAs and genes involved in ABA biosynthesis and signal transduction. **Table S11.** LncRNAs and genes involved in ETH biosynthesis and signal transduction. **Table S12.** LncRNAs and genes involved in starch and sucrose metabolism. **Table S13.** Dehydration response and transcriptional memory genes or lncRNAs in switchgrass, maize and *Arabidopsis.* (ZIP 3778 kb)

## Abbreviations

“-”: Downregulated significantly between A and B
“+”: Upregulated significantly between A and B
“=”: No significant change between A and B
AAO: Abscisic-aldehyde oxidase
ABA: Abscisic acid
ACO: Aminocyclopropane carboxylate oxidase
ACS: 1-aminocyclopropane-1-carboxylate synthase
AREBs/ABFs: ABA responsive element binding factors
BAM: Beta-amylases
BLAST: Basic local alignment search tool
C1: Control
D1: The first dehydration stress
D2: The secondary dehydration stress
EIN: Ethylene insensitive protein
ETH: Ethylene
FPKM: Fragments per kilobase of exon per million fragments mapped
GO: Gene ontology
HPLC-MS: High performance liquid chromatography - mass spectrometry
KEGG: Kyoto encyclopedia of genes and genomes
lncRNA: Long non-coding RNA
NCED: 9-cis-epoxycarotenoid dioxygenase
PHS: Plastidic alpha-glucan phosphorylase
PP2C: Protein phosphatase 2C
qRT-PCR: Quantitative real-time PCR
R1: First recovery
SnRK: Serine/threonine-protein kinase SRK
T-6-P: Trehalose-6-phosphate
TPS: Trehalose phosphate synthase
