# Supplementary material for: Long non-coding RNAs of switchgrass (Panicum virgatum L.) in multiple dehydration stresses
Source: BMC Plant Biol. 2018 May 4;18:79. doi: 10.1186/s12870-018-1288-3 (PMC5936019; doi:10.1186/s12870-018-1288-3)
Supplement: Supplementary file 1 — Figure S1. Overview of the RNA-Seq results and repeatability of different biological replicates. The first eight figures show the proportion of clean reads in the sequenced samples; the next three figures show the repeated expression of genes in two biological replicates; the last eight figures showed the correlations between differentially expressed genes in two biological replicates, and the distributions of upregulated and downregulated genes/lncRNAs in multiple dehydration stresses. C, control; D1–2, the first and second dehydration stresses; R1, the first recovery period. Figure S2. Water loss and survive rates in multiple dehydration stresses. A. Water loss from leaves during the first, second and third dehydration stresses. B. Survival rates of trained and non-trained switchgrass. Figure S3. Verification of four types of dehydration memory genes by quantitative real-time PCR. C, control; D1–2, first and second dehydration stresses; R1, first recovery period. Relative gene expressed levels were calculated using the ΔΔCT method with PveEF-1α as the internal control, and three biological replicates were performed for each experiment. (ZIP 1905 kb) [file 12870_2018_1288_MOESM1_ESM.zip › Fig.S1.pdf]

Fig.S1 The overview of RNA-Seq and repeatability of different biological replicates

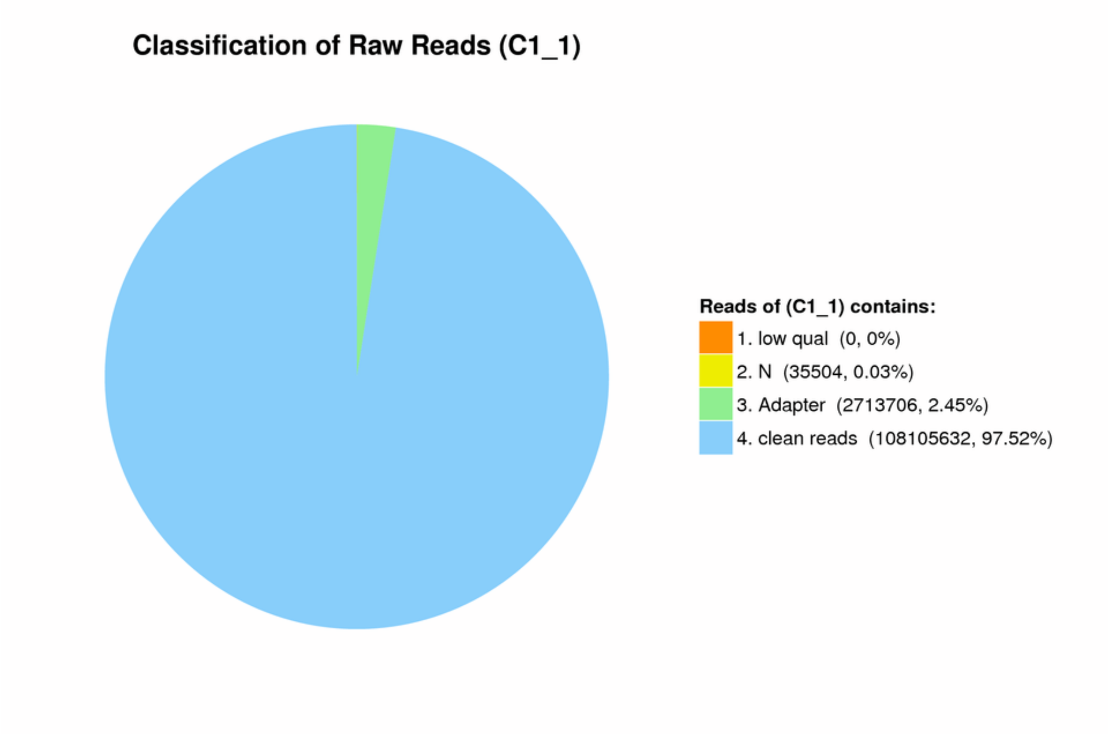

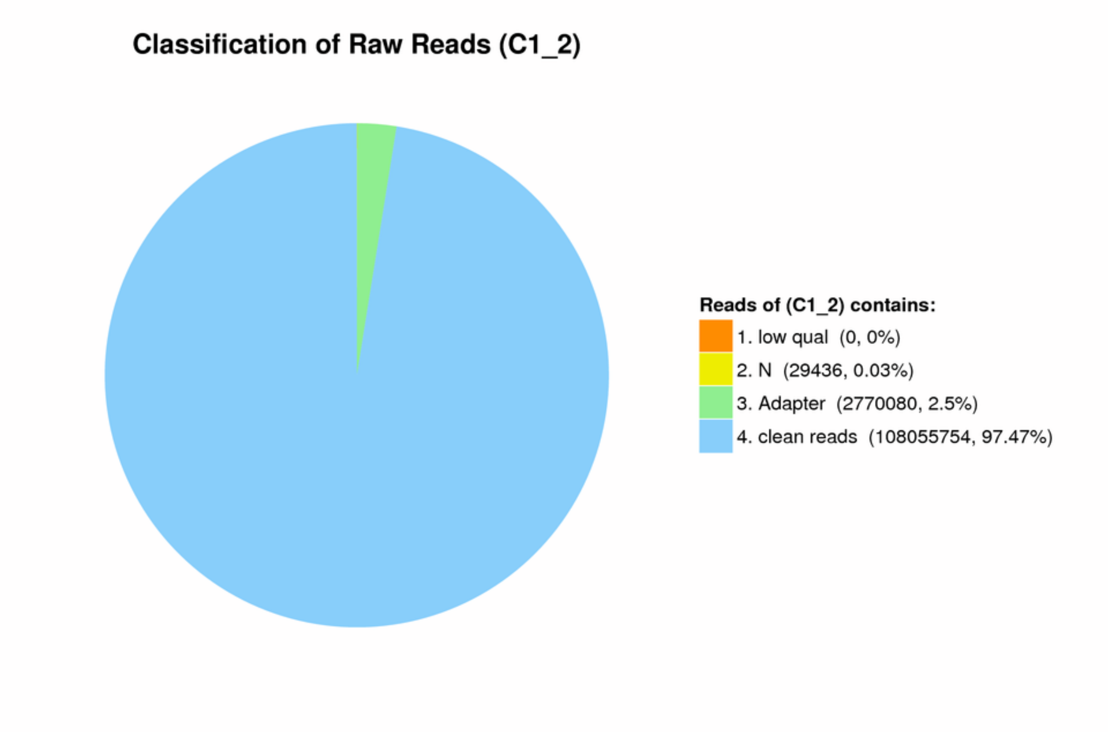

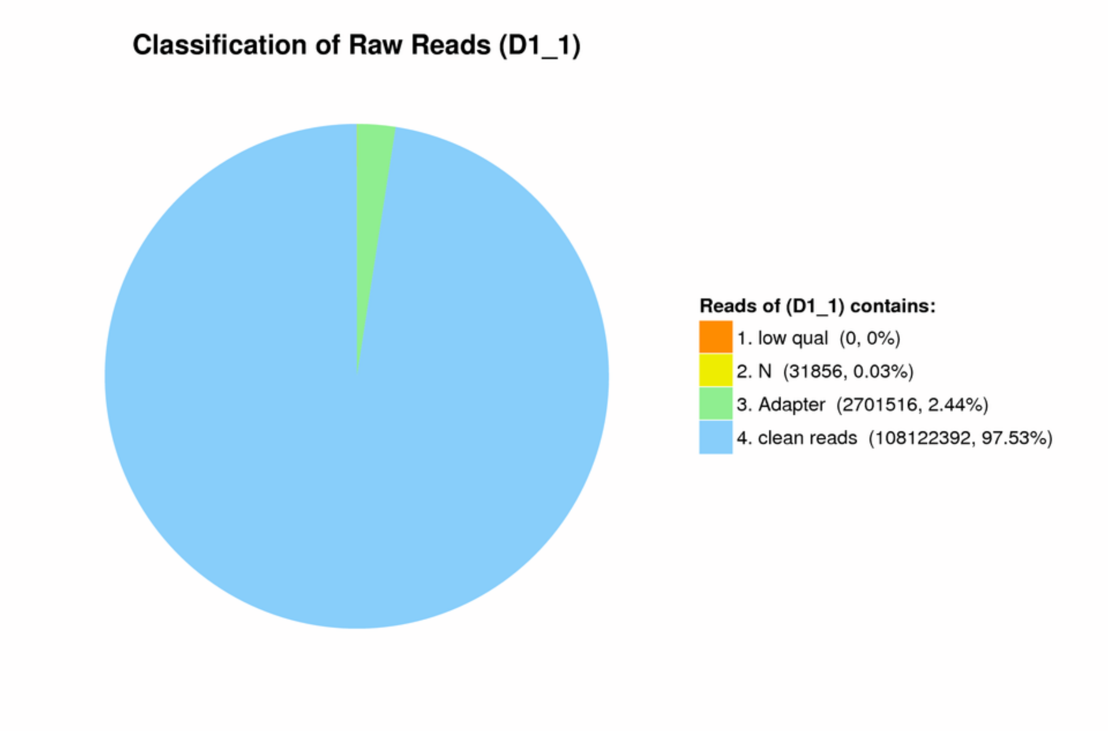
Classification of Raw Reads (D1_1)
Reads of (D1_1) contains:
1. low qual (0, 0%)
2. N (31856, 0.03%)
3. Adapter (2701516, 2.44%)
4. clean reads (108122392, 97.53%)

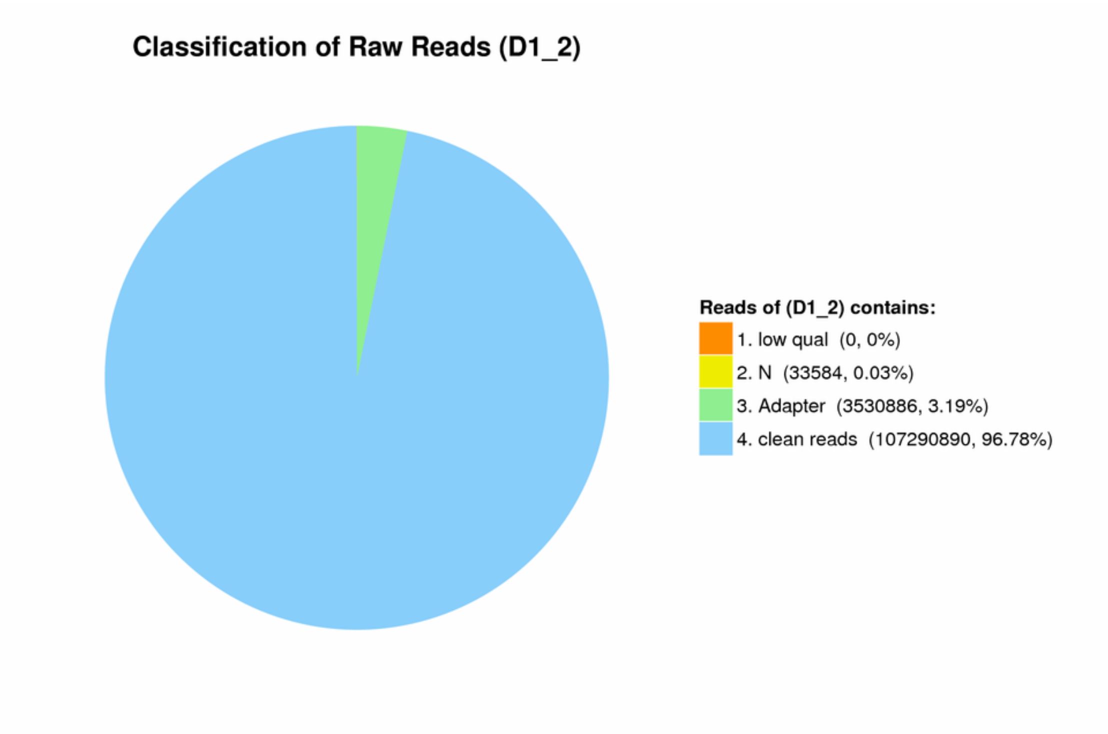
Classification of Raw Reads (D1_2)
Reads of (D1_2) contains:
1. low qual (0, 0%)
2. N (33584, 0.03%)
3. Adapter (3530886, 3.19%)
4. clean reads (107290890, 96.78%)

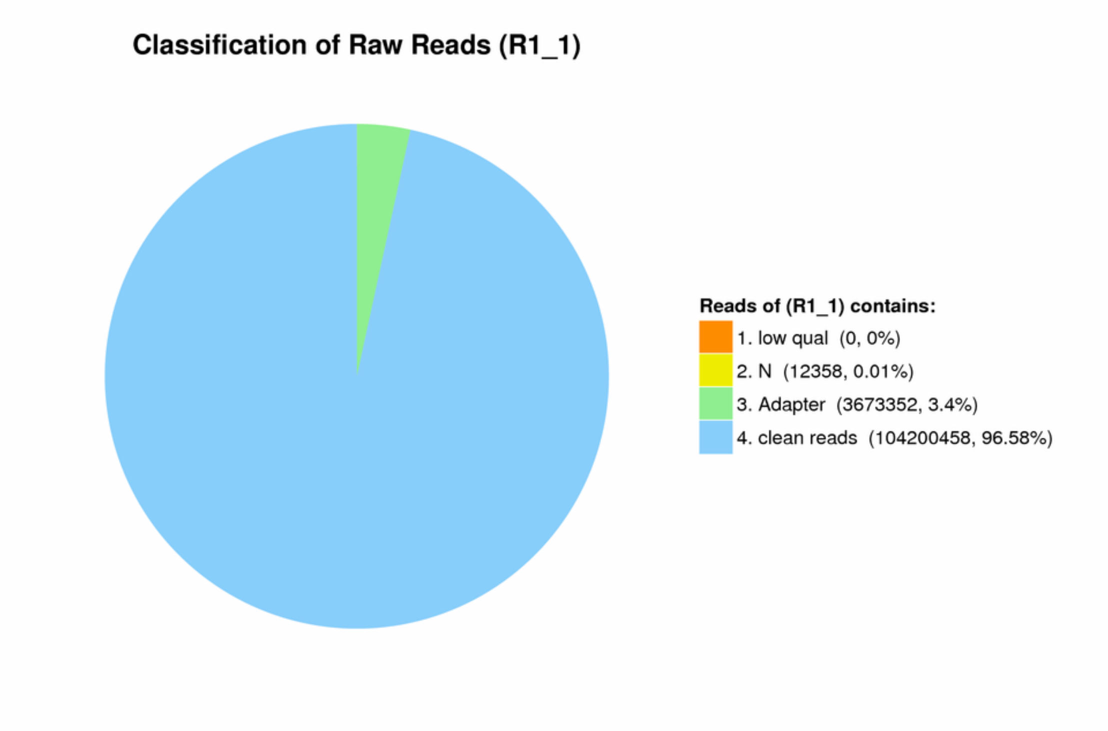
Classification of Raw Reads (R1_1)
Reads of (R1_1) contains:
1. low qual (0, 0%)
2. N (12358, 0.01%)
3. Adapter (3673352, 3.4%)
4. clean reads (104200458, 96.58%)

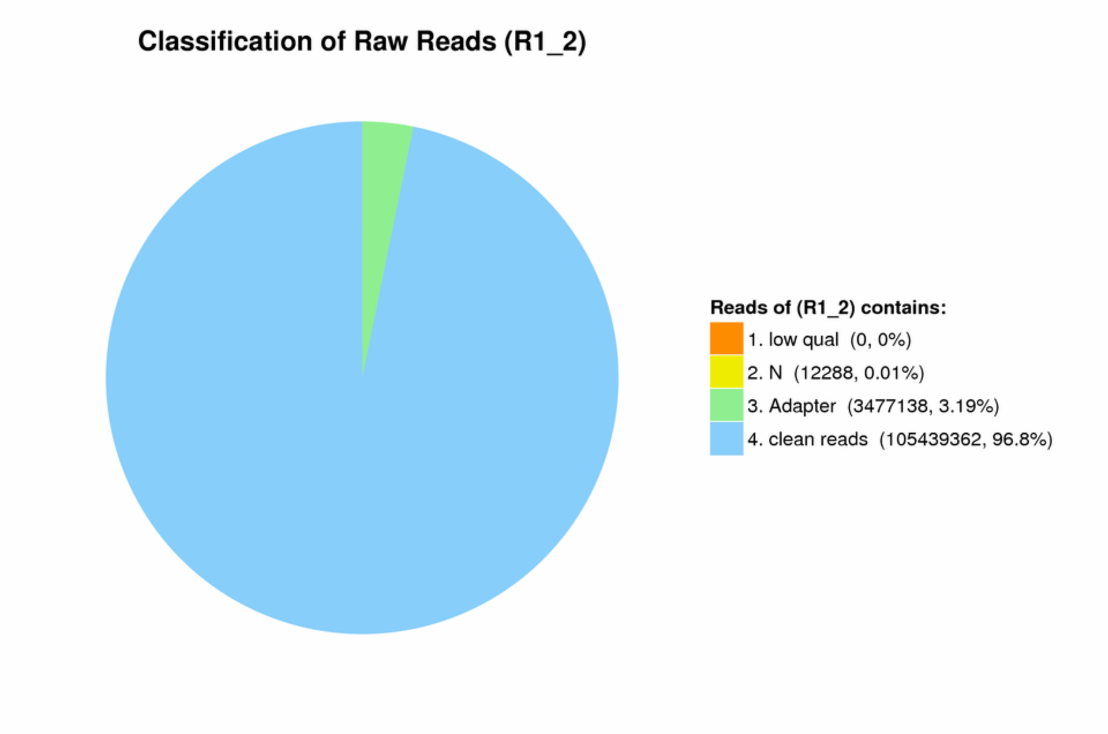
Classification of Raw Reads (R1_2)
Reads of (R1_2) contains:
1. low qual (0, 0%)
2. N (12288, 0.01%)
3. Adapter (3477138, 3.19%)
4. clean reads (105439362, 96.8%)

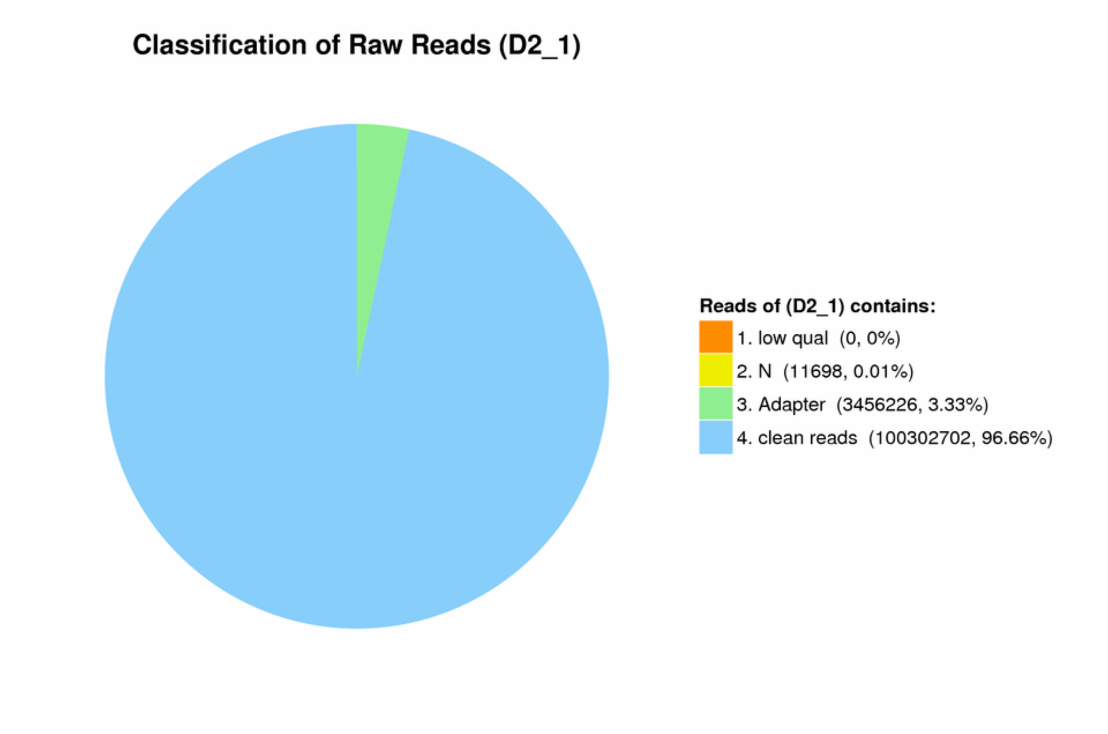

Classification of Raw Reads (D2_1)
Reads of (D2_1) contains:
1. low qual (0, 0%)
2. N (11698, 0.01%)
3. Adapter (3456226, 3.33%)
4. clean reads (100302702, 96.66%)

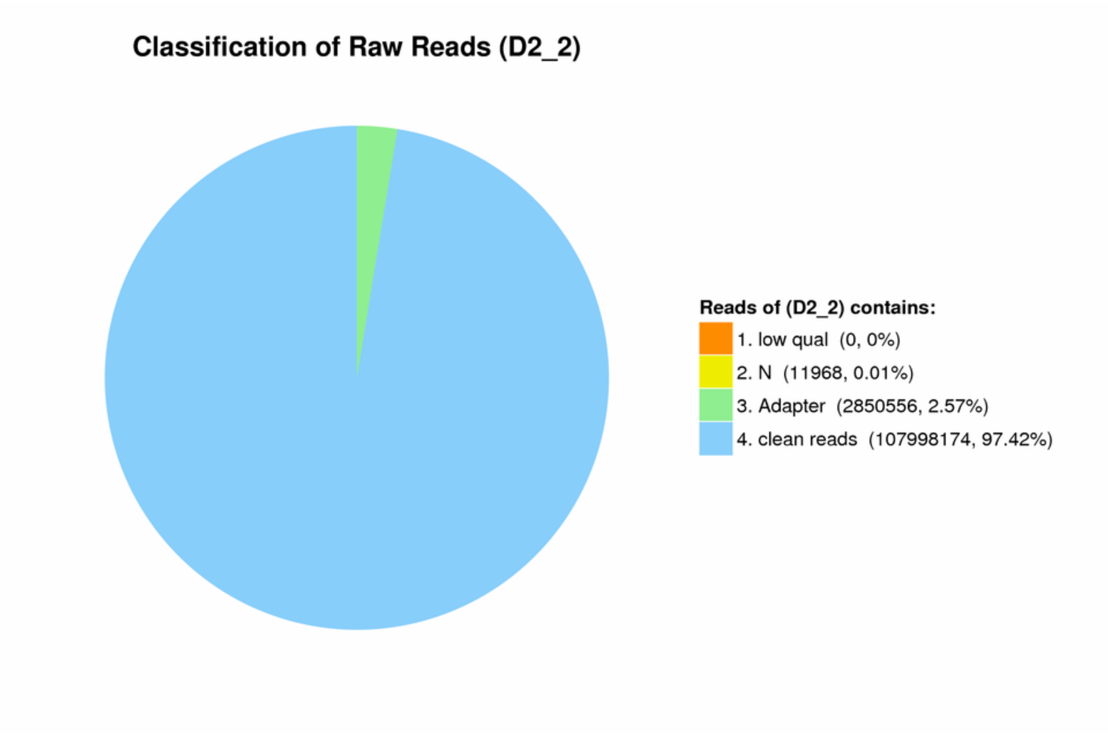

Classification of Raw Reads (D2_2)
Reads of (D2_2) contains:
1. low qual (0, 0%)
2. N (11968, 0.01%)
3. Adapter (2850556, 2.57%)
4. clean reads (107998174, 97.42%)

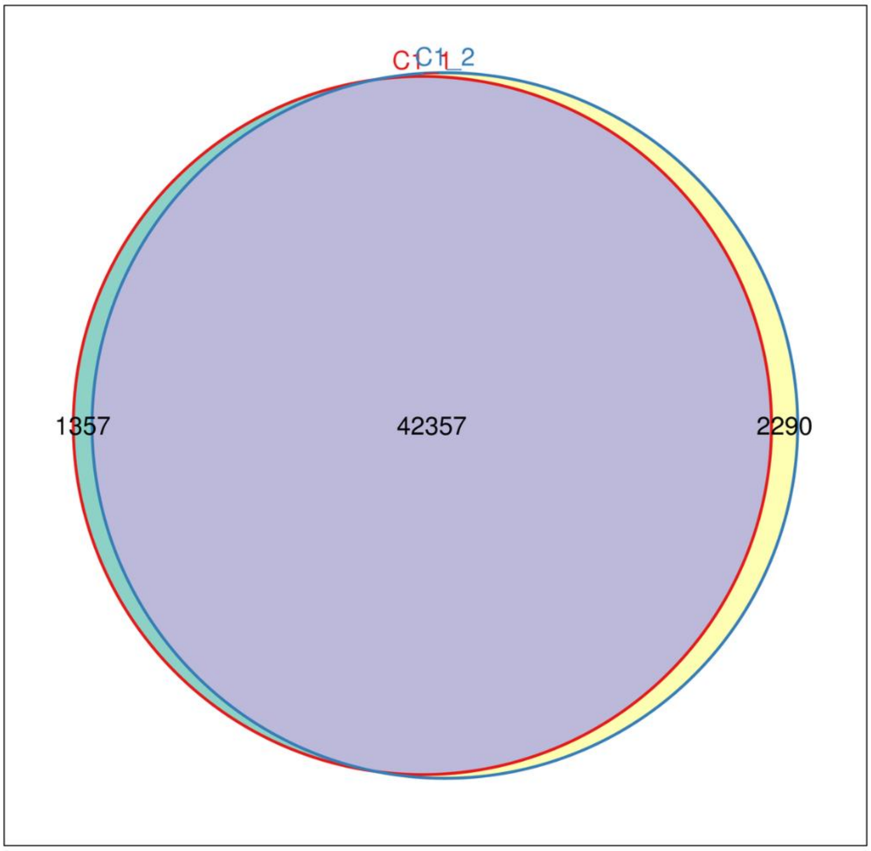
C1_1
C1_2
1357
42357
2290

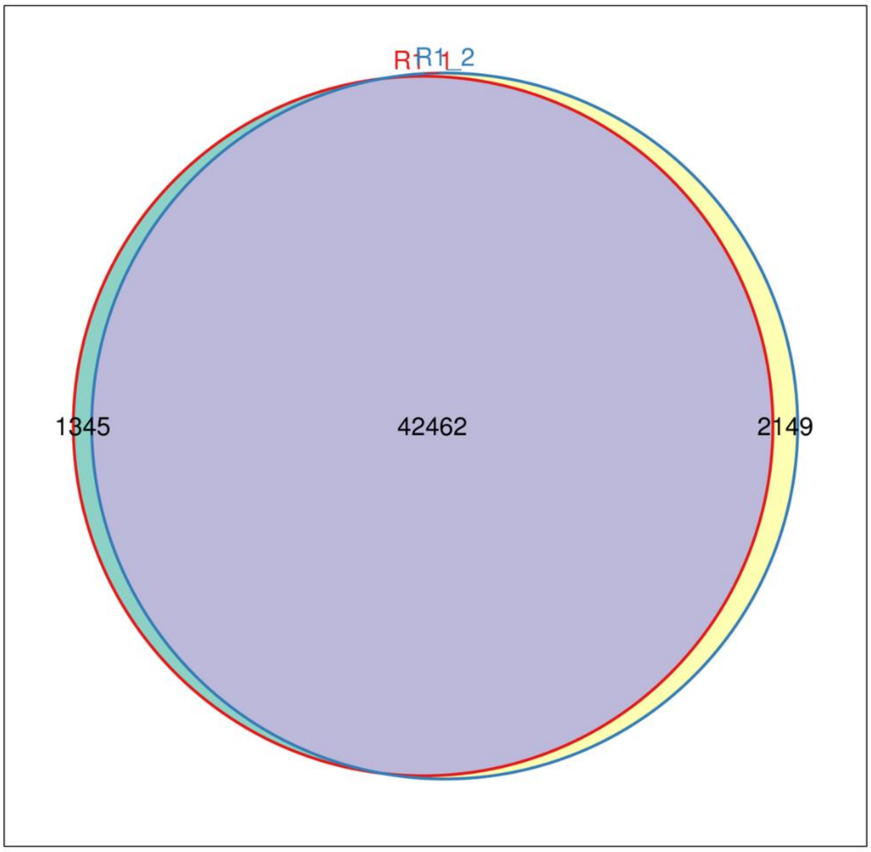
R1_1
R1_2
1345
42462
2149

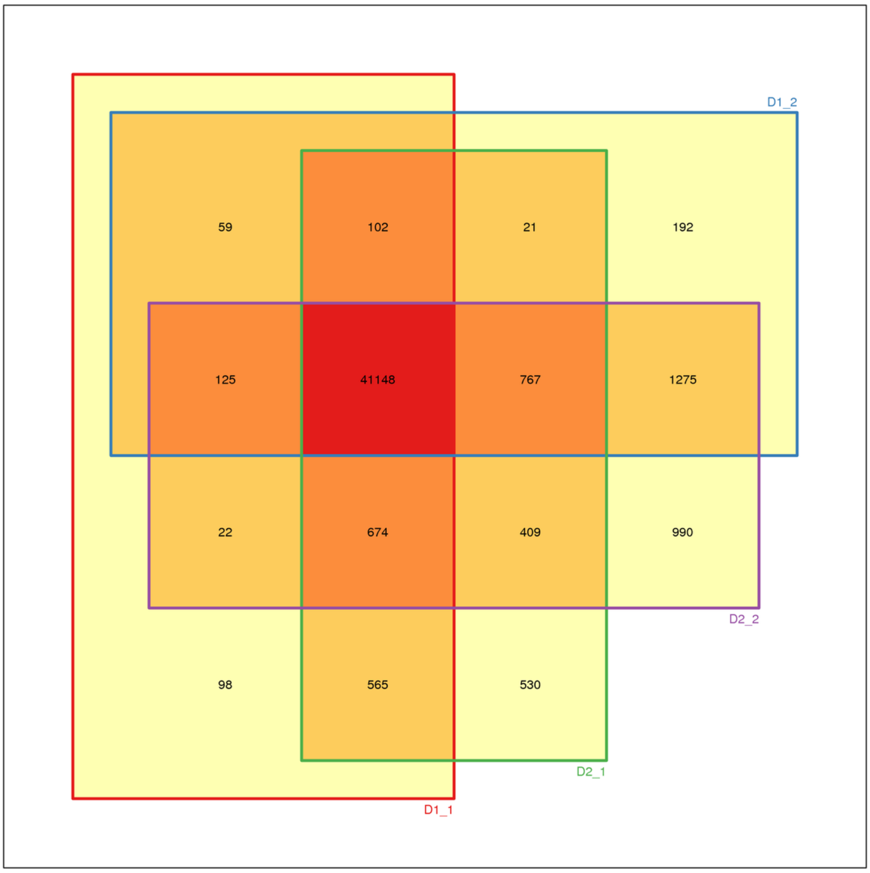
D1_2
59
102
21
192
125
41148
767
1275
22
674
409
990
D2_2
98
565
530
D2_1
D1_1

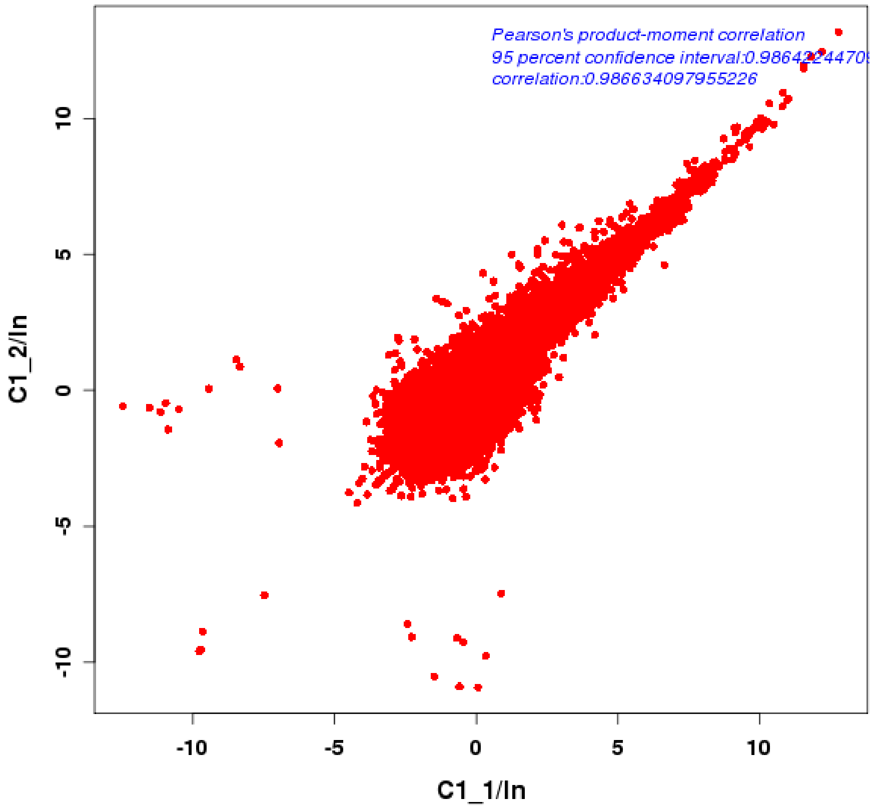
Pearson's product-moment correlation
95 percent confidence interval:0.98642244709
correlation:0.986634097955226
C1_2/ln
C1_1/ln
10
5
0
-5
-10

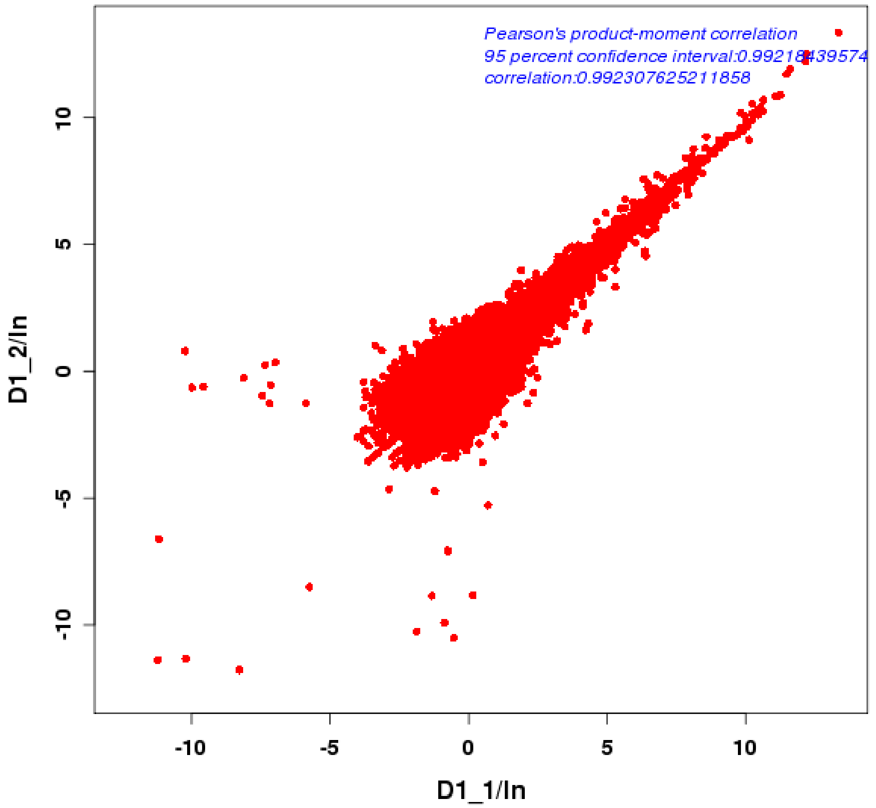

Pearson's product-moment correlation
95 percent confidence interval:0.99218439574
correlation:0.992307625211858
D1_2/In
D1_1/In

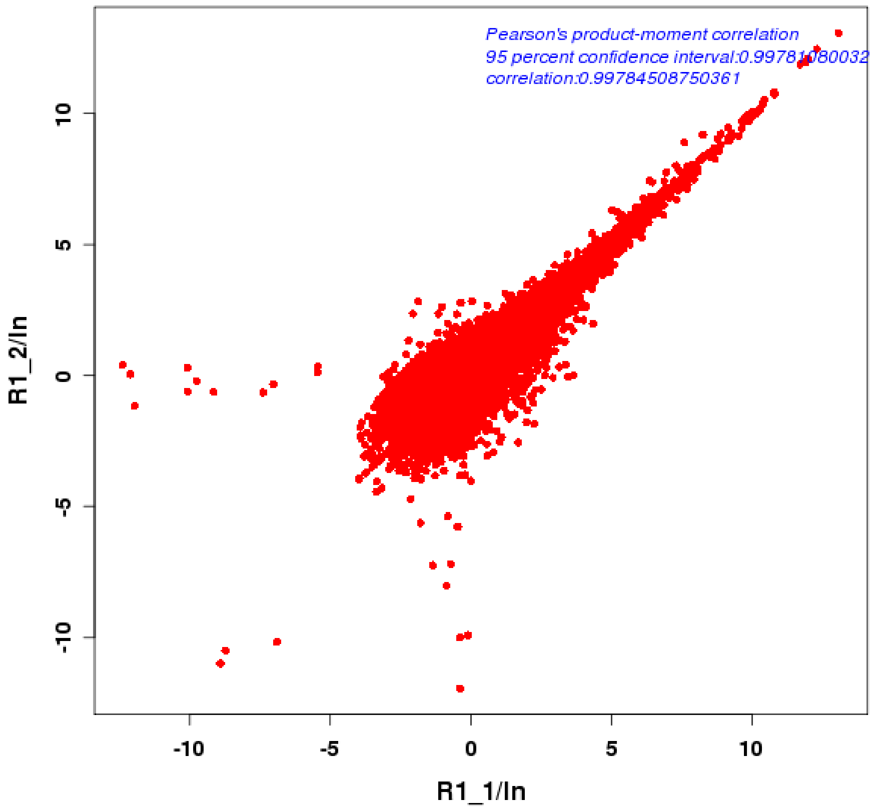

Pearson's product-moment correlation
95 percent confidence interval:0.99781080032
correlation:0.99784508750361
R1_2/In
R1_1/In

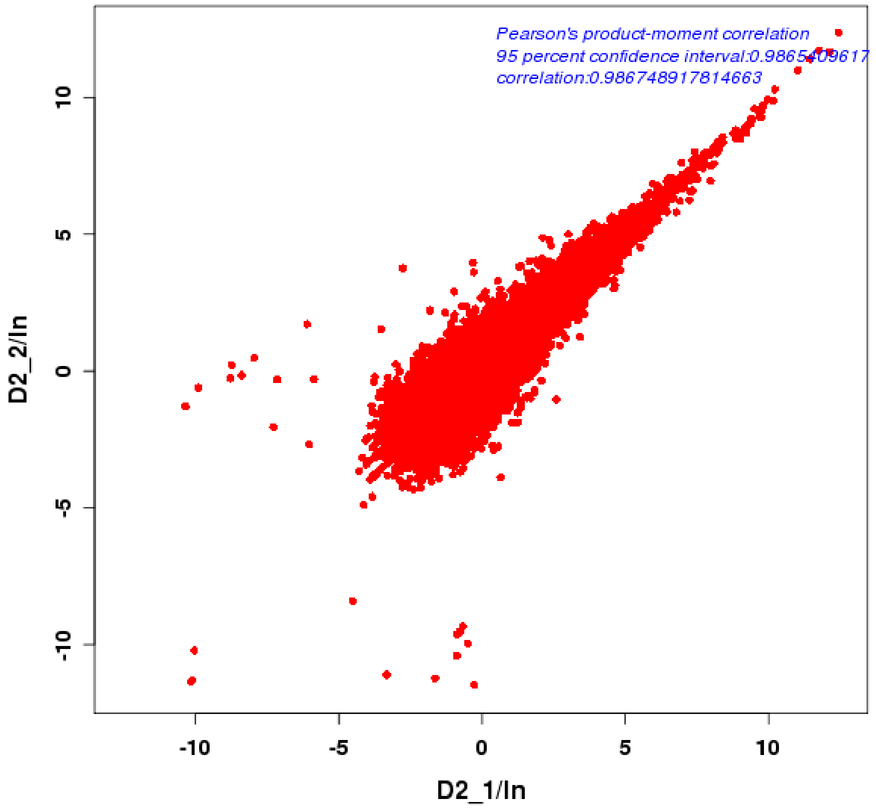

Gene Expression Level of C1 vs D1

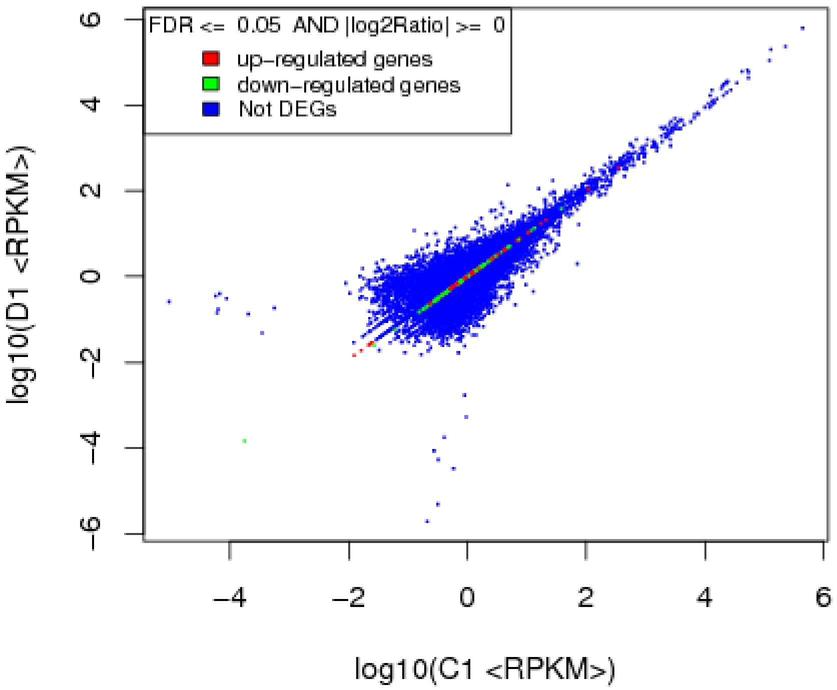

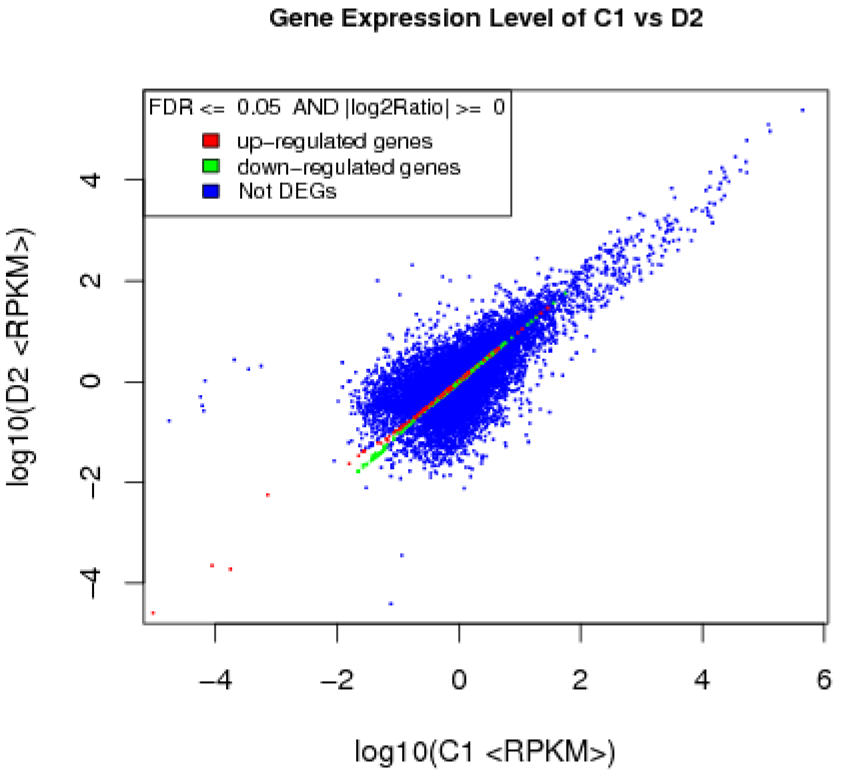
Gene Expression Level of C1 vs D2
FDR <= 0.05 AND |log2Ratio| >= 0
up-regulated genes
down-regulated genes
Not DEGs
log10(D2 <RPKM>)
log10(C1 <RPKM>)
4
2
0
-2
-4
-4
-2
0
2
4
6

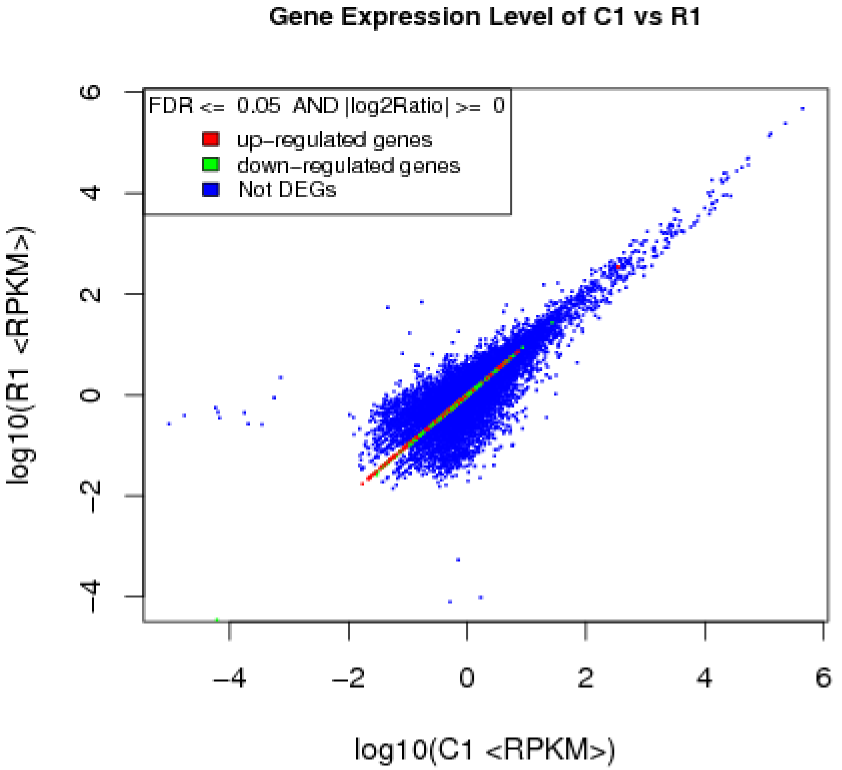
Gene Expression Level of C1 vs R1
FDR <= 0.05 AND |log2Ratio| >= 0
up-regulated genes
down-regulated genes
Not DEGs
log10(R1 <RPKM>)
log10(C1 <RPKM>)
6
4
2
0
-2
-4
-4
-2
0
2
4
6

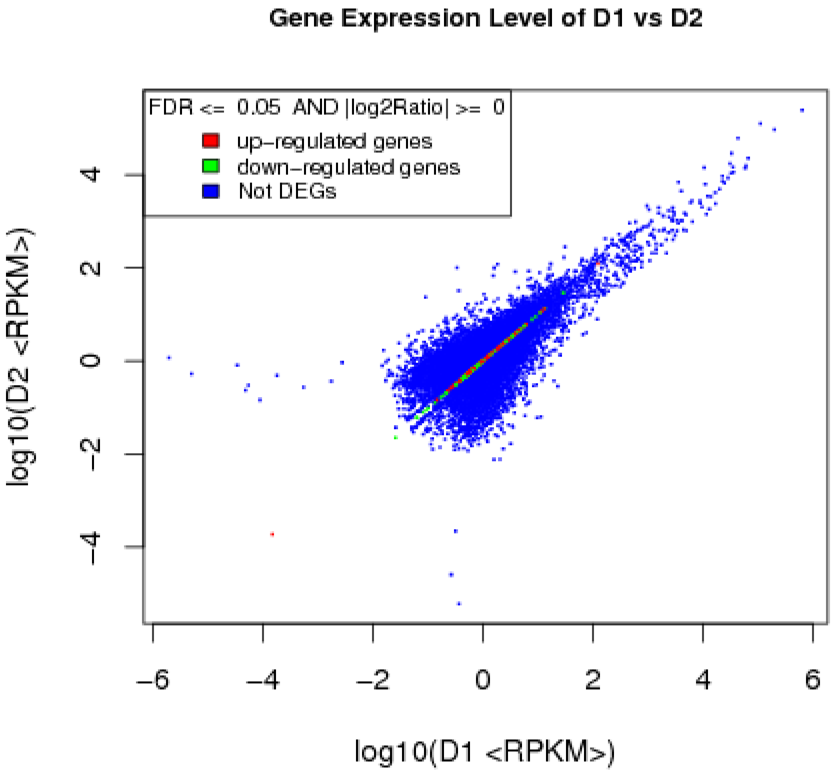
Gene Expression Level of D1 vs D2
FDR <= 0.05 AND |log2Ratio| >= 0
up−regulated genes
down−regulated genes
Not DEGs
log10(D2 <RPKM>)
log10(D1 <RPKM>)
−6
−4
−2
0
2
4
6
