# Supplementary figures and images for: Long non-coding RNAs of switchgrass (Panicum virgatum L.) in multiple dehydration stresses
Source: BMC Plant Biol. 2018 May 4;18:79. doi: 10.1186/s12870-018-1288-3 (PMC5936019; doi:10.1186/s12870-018-1288-3)

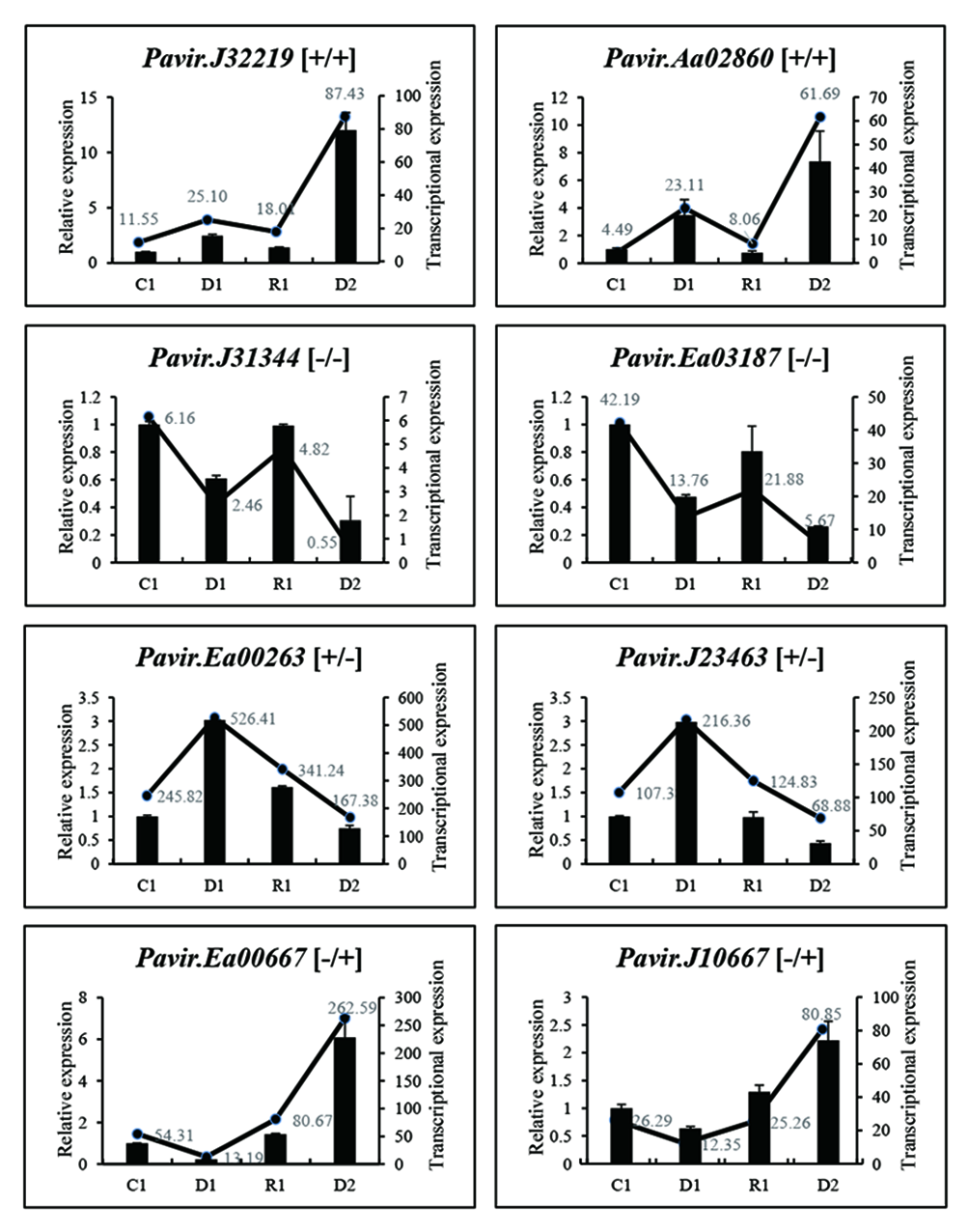

Supplement: Supplementary file 1 — Figure S1. Overview of the RNA-Seq results and repeatability of different biological replicates. The first eight figures show the proportion of clean reads in the sequenced samples; the next three figures show the repeated expression of genes in two biological replicates; the last eight figures showed the correlations between differentially expressed genes in two biological replicates, and the distributions of upregulated and downregulated genes/lncRNAs in multiple dehydration stresses. C, control; D1–2, the first and second dehydration stresses; R1, the first recovery period. Figure S2. Water loss and survive rates in multiple dehydration stresses. A. Water loss from leaves during the first, second and third dehydration stresses. B. Survival rates of trained and non-trained switchgrass. Figure S3. Verification of four types of dehydration memory genes by quantitative real-time PCR. C, control; D1–2, first and second dehydration stresses; R1, first recovery period. Relative gene expressed levels were calculated using the ΔΔCT method with PveEF-1α as the internal control, and three biological replicates were performed for each experiment. (ZIP 1905 kb) [file 12870_2018_1288_MOESM1_ESM.zip › Fig.S3.tif]

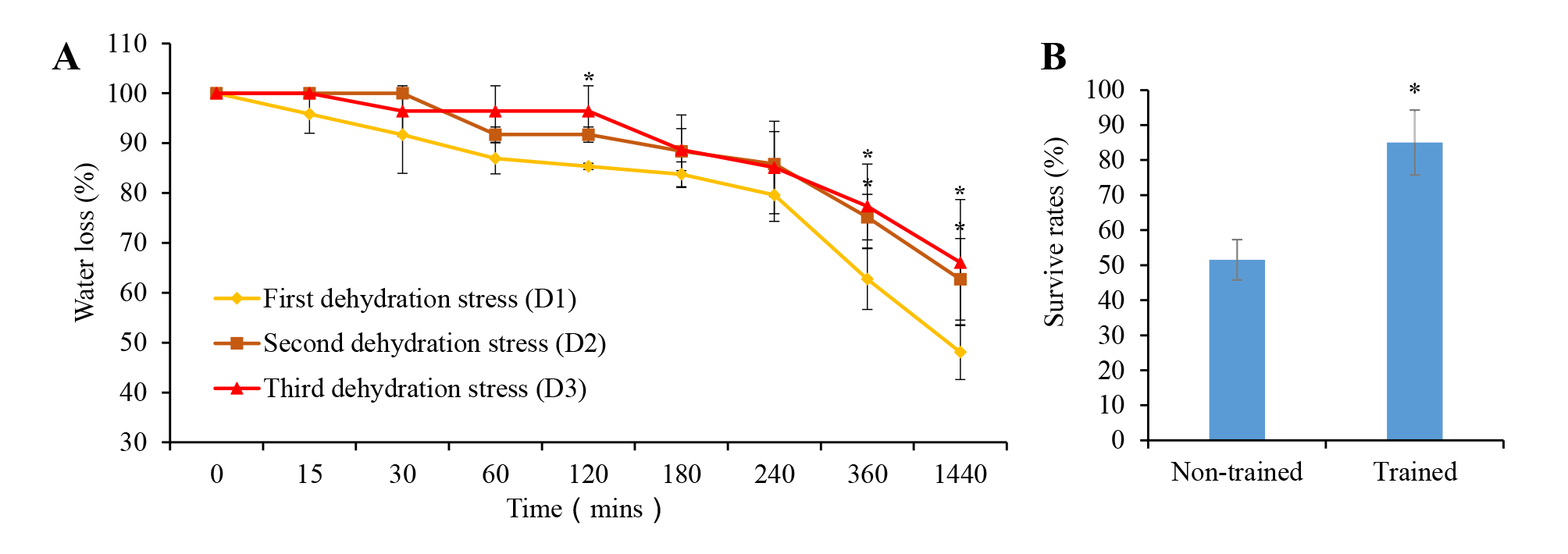

Supplement: Supplementary file 1 — Figure S1. Overview of the RNA-Seq results and repeatability of different biological replicates. The first eight figures show the proportion of clean reads in the sequenced samples; the next three figures show the repeated expression of genes in two biological replicates; the last eight figures showed the correlations between differentially expressed genes in two biological replicates, and the distributions of upregulated and downregulated genes/lncRNAs in multiple dehydration stresses. C, control; D1–2, the first and second dehydration stresses; R1, the first recovery period. Figure S2. Water loss and survive rates in multiple dehydration stresses. A. Water loss from leaves during the first, second and third dehydration stresses. B. Survival rates of trained and non-trained switchgrass. Figure S3. Verification of four types of dehydration memory genes by quantitative real-time PCR. C, control; D1–2, first and second dehydration stresses; R1, first recovery period. Relative gene expressed levels were calculated using the ΔΔCT method with PveEF-1α as the internal control, and three biological replicates were performed for each experiment. (ZIP 1905 kb) [file 12870_2018_1288_MOESM1_ESM.zip › Fig.S2.tif]
